# Pre-Existing Left Bundle Branch Block and Clinical Outcomes After Transcatheter Aortic Valve Replacement

**DOI:** 10.1016/j.jacasi.2023.11.007

**Published:** 2024-01-09

**Authors:** Tetsuya Saito, Taku Inohara, Hikaru Tsuruta, Fumiaki Yashima, Hideyuki Shimizu, Keiichi Fukuda, Yohei Ohno, Hidetaka Nishina, Masaki Izumo, Masahiko Asami, Toru Naganuma, Kazuki Mizutani, Masahiro Yamawaki, Norio Tada, Futoshi Yamanaka, Shinichi Shirai, Masahiko Noguchi, Hiroshi Ueno, Kensuke Takagi, Yusuke Watanabe, Masanori Yamamoto, Kentaro Hayashida, the OCEAN-TAVI Investigators

**Affiliations:** a Department of Cardiology, Keio University School of Medicine, Tokyo, Japan; b Department of Cardiology, Saiseikai Utsunomiya Hospital, Tochigi, Japan; c Department of Cardiovascular Surgery, Keio University School of Medicine, Tokyo, Japan; d Department of Cardiology, Tokai University School of Medicine, Isehara, Japan; e Department of Cardiology, Tsukuba Medical Center Hospital, Tsukuba, Japan; f Department of Cardiology, St. Marianna University, Tokyo, Japan; g Division of Cardiology, Mitsui Memorial Hospital, Tokyo, Japan; h Department of Cardiology, New Tokyo Hospital, Matsudo, Japan; i Department of Cardiology, Kinki University, Osaka, Japan; j Department of Cardiology, Saiseikai Yokohama-City Eastern Hospital, Tsurumi, Japan; k Department of Cardiology, Sendai Kousei Hospital, Sendai, Japan; l Department of Cardiology, Shonan Kamakura General Hospital, Kamakura, Japan; m Department of Cardiology, Kokura Memorial Hospital, Kokura, Japan; n Department of Cardiology, Tokyo Bay Urayasu Ichikawa Medical Center, Urayasu, Japan; o Department of Cardiology, Toyama University Hospital, Toyama, Japan; p Department of Cardiology, National Cerebral and Cardiovascular Center, Osaka, Japan; q Department of Cardiology, Teikyo University School of Medicine, Tokyo, Japan; r Department of Cardiology, Toyohashi Heart Center, Toyohashi, Japan; s Department of Cardiology, Nagoya Heart Center, Nagoya, Japan; t Department of Cardiology, Gifu Heart Center, Gifu, Japan

**Keywords:** left bundle branch block, propensity score, transcatheter aortic valve replacement

## Abstract

**Background:**

Few reports on pre-existing left bundle branch block (LBBB) in patients undergoing transcatheter aortic valve replacement (TAVR) are currently available. Further, no present studies compare patients with new onset LBBB with those with pre-existing LBBB.

**Objectives:**

This study aimed to investigate the association between pre-existing or new onset LBBB and clinical outcomes after TAVR.

**Methods:**

Using data from the Japanese multicenter registry, 5,996 patients who underwent TAVR between October 2013 and December 2019 were included. Patients were classified into 3 groups: no LBBB, pre-existing LBBB, and new onset LBBB. The 2-year clinical outcomes were compared between 3 groups using Cox proportional hazards models and propensity score analysis to adjust the differences in baseline characteristics.

**Results:**

Of 5,996 patients who underwent TAVR, 280 (4.6%) had pre-existing LBBB, while 1,658 (27.6%) experienced new onset LBBB. Compared with the no LBBB group, multivariable Cox regression analysis showed that pre-existing LBBB was associated not only with a higher 2-year all-cause (adjusted HR: 1.39; 95% CI: 1.06-1.82; *P =* 0.015) and cardiovascular (adjusted HR: 1.60; 95% CI: 1.04-2.48; *P =* 0.031) mortality, but also with higher all-cause (adjusted HR: 1.43, 95% CI: 1.07-1.91; *P =* 0.016) and cardiovascular (adjusted HR: 1.81, 95% CI:1.12-2.93; *P =* 0.014) mortality than the new onset LBBB group. Heart failure was the most common cause of cardiovascular death, with more heart failure deaths in the pre-existing LBBB group.

**Conclusions:**

Pre-existing LBBB was independently associated with poor clinical outcomes, reflecting an increased risk of cardiovascular mortality after TAVR. Patients with pre-existing LBBB should be carefully monitored.

Transcatheter aortic valve replacement (TAVR) is an established therapy for symptomatic severe aortic stenosis (AS).^1^^,^^2^ Preprocedural electrocardiography is particularly important to stratify the risks of patients undergoing TAVR. Pre-existing right bundle branch block (RBBB) is well recognized as a risk factor for permanent pacemaker implantation (PPI) after the procedure and increases the risks of all-cause and cardiovascular mortality.^3^^,^^4^ However, the prognostic impact of pre-existing left bundle branch block (LBBB) has not been well investigated.

Several studies have investigated the prognostic impact of new onset LBBB and yielded inconsistent results.^5^, ^6^, ^7^ The caveat is that these reports excluded patients with pre-existing LBBB, and only a few studies were conducted to investigate the impact of pre-existing LBBB on clinical outcomes. Fischer et al^8^ reported that pre-existing LBBB was a risk for early PPI after TAVR, but not for late PPI, and had no significant impact on mortality after TAVR. Given that the prognostic impact could be different between pre-existing and new onset LBBB, they should be separately treated in the analysis. Therefore, in the present study, we aimed to investigate the association with pre-existing or new onset LBBB and clinical outcomes in patients who underwent TAVR for severe AS.

## Methods

### Data source

We analyzed the data from the OCEAN-TAVI (Optimized transCathEter vAlvular iNtervention-transcatheter aortic valve implantation) registry.^9^ A total of 7,393 patients were enrolled in the OCEAN-TAVI registry between October 2013 and December 2019. The OCEAN-TAVI registry is a prospective, multicenter, observational registry of patients who underwent TAVR at 20 centers in Japan. The OCEAN-TAVI registry was registered with the University Hospital Medical Information Network Clinical Trial Registry and accepted by the International Committee of Medical Journal Editors (UMIN000020423). All study participants provided informed consent, and the registry was approved by the ethics committees of all participating institutions. Patients were followed up annually at the participating institutions. The events were site-reported from the participating institutions. To ensure consistency, the database was regularly audited by the data committee members.

### Study population

**Figure 1:**
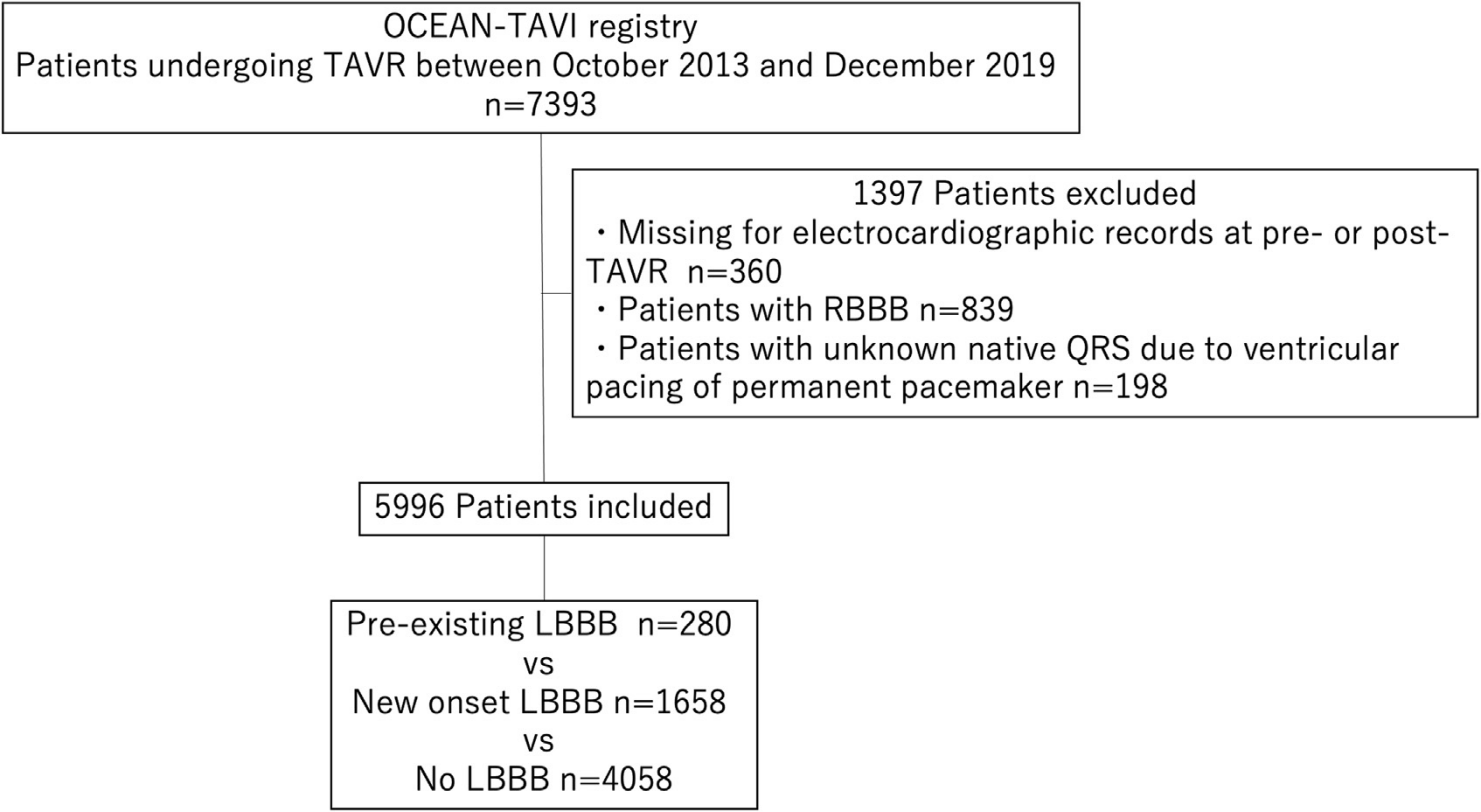
Study Flowchart Inclusion and exclusion criteria for the current study. LBBB = left bundle branch block; OCEAN-TAVI = Optimized transCathEter vAlvular iNtervention-transcatheter aortic valve implantation; RBBB = right bundle branch block; TAVR = transcatheter aortic valve replacement.

The study flow is presented in Figure 1. We excluded patients who had missing data for electrocardiographic records at either baseline or discharge (n = 360), patients with complete RBBB (n = 839), and patients with unknown native QRS due to ventricular pacing of a permanent pacemaker (n = 225). As a result, 5,996 patients were included in the analyses.

### Outcomes

The primary outcomes were 2-year all-cause cardiovascular and noncardiovascular mortality after TAVR. The secondary outcomes were death from heart failure, sudden cardiac death (SCD) after TAVR, 30-day mortality, and in-hospital complications. All-cause mortality, cardiovascular mortality, and complications were defined based on the Valve Academic Research Consortium-2 criteria.^10^ SCD was defined as death occurring within 1 hour of symptom onset if witnessed, or within the past 24 hours if not witnessed, according to the World Health Organization definition. Patients with confirmed sudden death due to terminal disease or noncardiac causes were not considered to have experienced SCD.^11^

### Electrocardiography and echocardiography

Twelve-lead electrocardiography and transthoracic echocardiography were performed at baseline, before hospital discharge, and at the annual follow-up. The diagnosis of intraventricular conduction disturbances was based on the American Heart Association/American College of Cardiology Foundation/Heart Rhythm Society recommendations for the standardization and interpretation of the electrocardiogram.^12^ As in previous reports,^3^^,^^4^^,^^8^ incomplete RBBB and LBBB were considered normal. All transthoracic echocardiographic parameters were measured according to American Society of Echocardiography guidelines.^13^^,^^14^ The degree of paravalvular leak (PVL) was measured in accordance with the guidelines and reported as a semiquantitative grade: none, trace, mild, moderate, or severe.^15^ Pre-existing LBBB was defined as the presence of complete LBBB on the electrocardiography at baseline. New onset LBBB was defined the presence of complete LBBB on postprocedural electrocardiography before hospital discharge.

### Statistical analysis

At first, baseline and 30-day outcomes and complications were compared between the 3 groups: pre-existing LBBB, new onset LBBB, and no LBBB (Table 1). Continuous variables were presented as median (Q1-Q3) and compared by analysis of variance tests. Categorical variables were presented as numbers and percentages and compared by the Pearson chi-square test or the Fisher exact test.

**Table 1:** Baseline Characteristics and 30-Day Outcomes

	Pre-Existing LBBB (n = 280)	New Onset LBBB (n = 1,658)	No LBBB (n = 4,058)	*P* Value
Preprocedural clinical data				
Age, y	85 (82-88)	85 (81-88)	84 (81-87)	0.001
Male	88 (31.4)	374 (22.6)	1,351 (33.3)	<0.001
Body mass index, kg/m^2^	21.5 (19.1-24.0)	22.2 (19.6-24.8)	22.1 (19.6-24.4)	0.072
Body surface area, m^2^	1.40 (1.30-1.53)	1.40 (1.30-1.50)	1.41 (1.30-1.58)	<0.001
NYHA functional class III or IV	154 (55.0)	615 (37.1)	1,658 (40.9)	<0.001
Hypertension	238 (85.0)	1,405 (84.7)	3,371 (83.1)	0.24
Dyslipidemia	150 (53.6)	941 (56.8)	2,245 (55.5)	0.47
Diabetes mellitus	80 (28.6)	451 (27.2)	1,089 (26.8)	0.8
Chronic kidney disease	212 (75.7)	1,162 (70.1)	2,774 (68.4)	0.023
Previous stroke	29 (10.4)	192 (11.6)	449 (11.1)	0.77
COPD	30 (10.7)	149 (9.0)	390 (9.9)	0.59
Peripheral artery disease	28 (10.0)	160 (9.7)	470 (11.6)	0.092
Coronary artery disease	95 (33.9)	505 (30.5)	1,352 (33.3)	0.099
Previous CABG	17 (6.1)	64 (3.9)	185 (4.6)	0.2
Atrial fibrillation	57 (20.4)	322 (19.4)	856 (21.1)	0.36
Permanent pacemaker	25 (8.9)	23 (1.4)	130 (3.2)	<0.001
Clinical frailty scale	4 (3-5)	4 (3-4)	4 (3-4)	0.096
Beta-blockers	102 (36.4)	555 (33.5)	1,333 (32.8)	0.45
RAS inhibitors	145 (51.8)	899 (54.2)	2,089 (51.5)	0.17
STS risk score, %	7.20 (4.94-11.6)	6.04 (4.21-8.84)	6.14 (4.18-9.20)	<0.001
Nontransfemoral approach	25 (8.9)	111 (6.7)	433 (10.7)	<0.001
Local anesthesia	101 (36.1)	628 (37.9)	1,468 (36.2)	0.47
Hemoglobin, g/dL	11.3 (10.4-12.4)	11.3 (10.2-12.5)	11.4 (10.3-12.5)	0.67
eGFR, mL/min/1.73 m^2^	48.0 (36.9-59.1)	50.6 (38.5-62.7)	51.0 (38.5-64.0)	0.076
Albumin <3.5 g/dL	75 (27.1)	395 (23.9)	976 (24.2)	0.51
BNP, pg/mL	380 (175-782)	209 (93-455)	236 (102-526)	<0.001
BNP ≥400 pg/mL or NT-proBNP ≥1,600 pg/mL	148 (54.4)	556 (34.0)	1,532 (38.3)	<0.001
Preprocedural echocardiographic data				
Aortic valve area, cm^2^	0.61 (0.49-0.72)	0.63 (0.51-0.77)	0.63 (0.50-0.75)	0.022
Peak velocity, m/s	4.30 (3.70-4.80)	4.50 (4.04-5.08)	4.50 (4.06-5.10)	<0.001
Mean pressure gradient, mm Hg	42.0 (31.3-54.0)	47.0 (36.9-61.0)	47.0 (37.5-61.0)	<0.001
LV end-diastolic dimension, mm	46.0 (41.0-51.0)	42.4 (39.0-46.6)	43.8 (40.0-48.0)	<0.001
LV end-systolic dimension, mm	32.0 (27.5-40.3)	27.0 (24.0-31.0)	28.0 (24.0-33.0)	<0.001
LVEF, %	51.0 (38.8-61.0)	64.0 (56.7-68.9)	62.7 (53.7-68.0)	<0.001
Aortic regurgitation ≥moderate	35 (12.5)	144 (8.7)	442 (10.9)	0.022
Mitral regurgitation ≥moderate	49 (17.5)	156 (9.4)	465 (11.5)	<0.001
Tricuspid regurgitation ≥moderate	30 (10.7)	140 (8.4)	329 (8.1)	0.31
Procedural data				
Nontransfemoral approach	25 (8.9)	111 (6.7)	433 (10.7)	<0.001
Local anesthesia	101 (36.1)	628 (37.9)	1,468 (36.2)	0.47
Balloon expandable valves	218 (77.9)	1,159 (69.9)	3,192 (78.7)	<0.001
Postprocedural outcomes and complications				
30-d mortality	4 (1.4)	23 (1.4)	49 (1.2)	0.83
Stroke	6 (2.1)	33 (2.0)	105 (2.6)	0.39
Myocardial infarction	3 (1.1)	5 (0.3)	26 (0.6)	0.15
Vascular complications	25 (8.9)	112 (6.8)	308 (7.6)	0.33
AKI	29 (10.4)	123 (7.4)	331 (8.2)	0.22
Bleeding	35 (12.5)	198 (11.9)	592 (14.6)	0.025
New pacemaker	8 (2.9)	92 (5.5)	124 (3.1)	<0.001
Indexed EOA, cm^2^/m^2^	1.12 (0.94-1.33)	1.15 (0.97-1.38)	1.14 (0.96-1.35)	0.039
THV mean pressure gradient, mm Hg	10.0 (7.9-13.9)	10.0 (7.4-13.1)	10.5 (8.0-14.0)	0.003
PVL ≥moderate	7 (2.5)	34 (2.1)	77 (1.9)	0.74

Values are median (Q1-Q3) or n (%).

AKI = acute kidney injury; BNP = B-type natriuretic peptide; CABG = coronary artery bypass grafting; COPD = chronic obstructive pulmonary disease; eGFR = estimated glomerular filtration rate; EOA = effective orifice area; LBBB = left bundle branch block; LV = left ventricular; LVEF = left ventricular ejection fraction; NT-proBNP = N-terminal pro–B-type natriuretic peptide; PVL = paravalvular leak; RAS = renin-angiotensin system; STS = Society of Thoracic Surgeons; THV = transcatheter heart valve.

The cumulative incidences of all-cause, cardiovascular, and noncardiovascular mortality; death from heart failure; and SCD were calculated using the Kaplan-Meier method. Log-rank test and Cox regression analyses were performed. We checked the proportional hazard assumptions of each outcome using Schoenfeld residuals (Supplemental Figure 1). Univariable Cox regression analyses were performed for 2-year clinical outcomes. Thereafter, multivariable analyses were performed to examine variables that were independently associated with all-cause, cardiovascular, and noncardiovascular mortality, as well as death from heart failure and SCD. In multivariable analysis, variables associated with mortality, based on previous studies, were included.^16^, ^17^, ^18^, ^19^, ^20^, ^21^, ^22^ The variables included in the multivariate analysis of mortality were following: age, sex, body surface area, NYHA functional class III or IV, hypertension, dyslipidemia, diabetes mellitus, chronic kidney disease, previous stroke, chronic obstructive pulmonary disease, peripheral artery disease, liver disease, coronary artery disease, previous coronary artery bypass grafting, atrial fibrillation, permanent pacemaker, clinical frailty score, nontransfemoral approach, local anesthesia, hemoglobin, albumin <3.5 g/dL, B-type natriuretic peptide (BNP) ≥400 pg/mL or N-terminal pro-BNP ≥1,600 pg/mL, left ventricular ejection fraction (LVEF) ≤40%, mitral regurgitation (MR) ≥moderate, tricuspid regurgitation ≥moderate, stroke, myocardial infarction, vascular complications, acute kidney injury, bleeding, new pacemaker implantation, and PVL ≥moderate.

To ensure adequately robust results, propensity score analysis was performed. The propensity score was calculated using a multinomial logistic model to estimate the probabilities of pre-existing LBBB, new onset LBBB, and no LBBB.^23^ The covariables included in the multinomial logistic model are listed in Supplemental Table 1. The covariates included were the following: age, sex, body mass index, body surface area, NYHA functional class III or IV, hypertension, dyslipidemia, diabetes mellitus, chronic kidney disease, previous stroke, chronic obstructive pulmonary disease, peripheral artery disease, liver disease, coronary artery disease, previous coronary artery bypass grafting, atrial fibrillation, permanent pacemaker, clinical frailty score, beta-blockers, renin-angiotensin system inhibitors, nontransfemoral approach, local anesthesia, hemoglobin, estimated glomerular filtration rate, albumin <3.5 g/dL, BNP ≥400 pg/mL or N-terminal pro-BNP ≥1,600 pg/mL, LVEF, aortic regurgitation ≥moderate, MR ≥moderate, tricuspid regurgitation ≥moderate, balloon expandable valve, stroke, myocardial infarction, vascular complications, acute kidney injury, bleeding, new pacemaker implantation, transcatheter heart valve mean pressure gradient, indexed effective orifice area, and PVL ≥moderate. Because of the difficulty of propensity score matching for the 3 groups,^24^ the inverse probability of treatment weighting (IPTW) method was used to analyze 2-year outcomes among the 3 groups. We used truncated weights at the 5th and 95th percentiles to exclude the influence of extreme weights in the weighted cohort. Balance between 3 groups was assessed using the maximum absolute standardized mean difference.

Subgroup analyses were performed because of large differences in baseline characters. Subgroup analyses for all-cause mortality were performed for age (≥85 or <85 years), sex, NYHA functional class (I, II or III, IV), and LVEF (≥50% or <50 %). Interaction tests between each covariate were performed.

To address the issue of residual confounding, sensitivity analyses using an array approach were performed to investigate how the observed HR may be affected by certain variations in the assumptions about the presence of an unmeasured confounder.^25^ This method is a statistical technique used to adjust for confounding factors that are difficult to adjust for or measure due to the nature of observational studies.^26^ The observed HRs for pre-existing LBBB using new onset LBBB as a reference were 1.43 for all-cause mortality and 1.81 for cardiovascular mortality. Prevalence of the unmeasured confounder in new onset LBBB group was set at 0.5. Two factors were varied: the strength of the unmeasured confounder-outcome association (1.0-5.5) and the prevalence of the unmeasured confounder in the pre-existing LBBB group (0.0-1.0). A 3-dimensional mesh plot was constructed to check the impacts on the fully adjusted HR by varied unmeasured confounder settings.

Finally, the post hoc analysis with the 2-arm comparison was added to confirm the consistency of the results found in the 3-arm comparison. Multivariate Cox regression and IPTW analyses were performed for all-cause mortality in each of the 2 groups.

The loss of cases due to missing values in the multivariable analyses and propensity score analysis was 2.3%. The analysis of the pattern of missing values is shown in Supplemental Figure 2. Because the proportion of missing values was small, multiple imputation was not performed. All statistical analyses were performed using R software version 3.6.1 (R Foundation for Statistical Computing). All tests were 2-sided, and *P* values of <0.05 were considered statistically significant.

## Results

### Baseline characteristics and 30-day outcomes

The baseline characteristics and 30-day outcomes of the study are shown in Table 1. Among 5,996 patients, baseline electrocardiography showed LBBB in 280 (4.6%). New onset LBBB occurred in 1,658 (27.6%) patients. Patients with pre-existing LBBB were more symptomatic and had worse renal functions, higher BNP, larger left ventricular diameters, lower LVEF, and more severe MR, resulting in higher Society of Thoracic Surgeons scores. Few patients with new onset LBBBs were treated with the balloon expandable valve. Postprocedural bleeding and new pacemaker implantation were more common in patients with new onset LBBB. The analysis with respect to the comparison of patients included in the study with those excluded is presented in Supplemental Table 2.

### Mortality

The Kaplan-Meier curves of all-cause, cardiovascular, and noncardiovascular mortality for the 3 groups of pre-existing LBBB, new onset LBBB, and no LBBB are shown in the Central Illustration. The median follow-up period was 686 days (Q1-Q3: 372-744 days). During the follow-up period, 988 patients died of all-cause, 321 of cardiovascular, and 667 of noncardiovascular causes. There was a significant difference among the 3 groups in 2-year all-cause (log-rank *P =* 0.0074) and cardiovascular (log-rank *P =* 0.0012) mortality. However, there was no significant difference in noncardiovascular mortality between the 3 groups (log-rank *P =* 0.13). In the multivariable Cox analysis, patients with pre-existing LBBB had higher all-cause (adjusted HR: 1.39; 95% CI: 1.06-1.82; *P* = 0.015) and cardiovascular (adjusted HR: 1.60; 95% CI: 1.04-2.48; *P* = 0.031) mortality than those without LBBB, and even higher all-cause (adjusted HR: 1.43; 95% CI: 1.07-1.91; *P* = 0.016) and cardiovascular (adjusted HR: 1.81; 95% CI: 1.12-2.93; *P* = 0.014) mortality than those with new onset LBBB. New onset LBBB was not associated with all-cause (adjusted HR: 0.97; 95% CI: 0.83-1.13; *P* = 0.75) or cardiovascular mortality (adjusted HR: 0.88; 95% CI: 0.66-1.17; *P* = 0.39) (Table 2). Multivariate analysis of noncardiac deaths showed no significant differences among the 3 groups. The full univariable and multivariable model results are shown in Supplemental Tables 3 to 5.

**Central Illustration:**
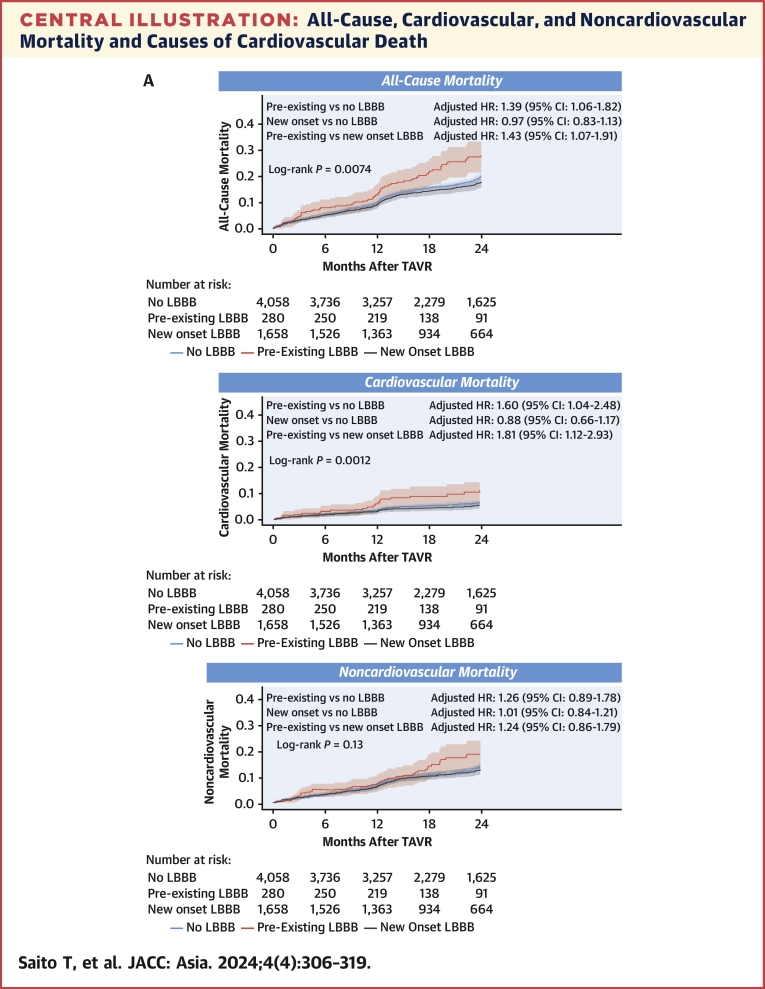

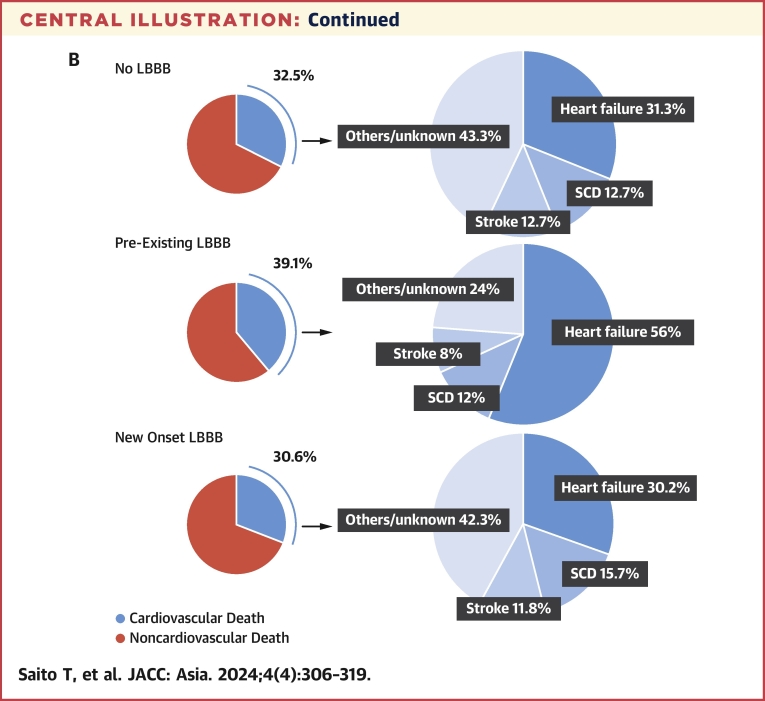
All-Cause, Cardiovascular, and Noncardiovascular Mortality and Causes of Cardiovascular Death (A) All-cause and cardiovascular mortality of patients with left bundle branch block (LBBB) compared with those without in overall cohort. (B) Causes of cardiovascular death in no LBBB, pre-existing LBBB, and new onset LBBB groups. SCD = sudden cardiac death.

**Table 2:** Results of Primary Outcomes Analyses

	Number of Events/N	Vs No LBBB		Vs New Onset LBBB	
		HR (95% CI)	*P* Value	HR (95% CI)	*P* Value
Unadjusted					
All-cause mortality					
No LBBB	676/4,058	Reference		1.11 (0.96-1.29)	0.13
Pre-existing LBBB	64/280	1.44 (1.12-1.87)	0.004	1.61 (1.22-2.12)	0.001
New onset LBBB	248/1,658	0.89 (0.77-1.03)	0.13	Reference	
Cardiovascular mortality					
No LBBB	220/4,058	Reference		1.18 (0.91-1.54)	0.19
Pre-existing LBBB	25/280	1.72 (1.14-2.60)	0.01	2.04 (1.29-3.21)	0.001
New onset LBBB	76/1,658	0.84 (0.65-1.09)	0.19	Reference	
Noncardiovascular mortality					
No LBBB	456/4,058	Reference		1.08 (0.91-1.29)	0.34
Pre-existing LBBB	39/280	1.31 (0.94-1.81)	0.11	1.42 (1.00-2.01)	0.045
New onset LBBB	172/1,658	0.91 (0.77-1.09)	0.34	Reference	
Multivariable Cox regression analyses					
All-cause mortality					
No LBBB	676/4,058	Reference		1.02 (0.87-1.19)	0.75
Pre-existing LBBB	64/280	1.39 (1.06-1.82)	0.015	1.43 (1.07-1.91)	0.016
New onset LBBB	248/1,658	0.97 (0.83-1.13)	0.75	Reference	
Cardiovascular mortality					
No LBBB	220/4,058	Reference		1.13 (0.85-1.50)	0.39
Pre-existing LBBB	25/280	1.60 (1.04-2.48)	0.031	1.81 (1.12-2.93)	0.014
New onset LBBB	76/1,658	0.88 (0.66-1.17)	0.39	Reference	
Noncardiovascular mortality					
No LBBB	456/4,058	Reference		0.99 (0.82-1.19)	0.89
Pre-existing LBBB	39/280	1.26 (0.89-1.78)	0.19	1.24 (0.86-1.79)	0.24
New onset LBBB	172/1,658	1.01 (0.84-1.21)	0.89	Reference	
IPTW analyses					
All-cause mortality					
No LBBB	890/5,685	Reference		1.06 (0.90-1.24)	0.46
Pre-existing LBBB	318/1,480	1.44 (1.09-1.90)	0.01	1.52 (1.13-2.06)	0.005
New onset LBBB	820/5,567	0.94 (0.80-1.10)	0.46	Reference	
Cardiovascular mortality					
No LBBB	273/5,685	Reference		1.14 (0.85-1.54)	0.35
Pre-existing LBBB	136/1,480	1.99 (1.28-3.07)	0.001	2.28 (1.40-3.70)	0.001
New onset LBBB	233/5,567	0.87 (0.64-1.16)	0.35	Reference	
Noncardiovascular mortality					
No LBBB	617/5,685	Reference		1.02 (0.84-1.23)	0.79
Pre-existing LBBB	182/1,480	1.19 (0.82-1.72)	0.34	1.22 (0.83-1.81)	0.3
New onset LBBB	587/5,567	0.97 (0.80-1.17)	0.79	Reference	

The variables included in the multivariate Cox regression analyses were following: age, sex, body surface area, NYHA functional class III or IV, hypertension, dyslipidemia, diabetes mellitus, chronic kidney disease, previous stroke, chronic obstructive pulmonary disease, peripheral artery disease, liver disease, coronary artery disease, previous coronary artery bypass grafting, atrial fibrillation, permanent pacemaker, clinical frailty score, nontransfemoral approach, local anesthesia, hemoglobin, albumin <3.5 g/dL, B-type natriuretic peptide ≥400 pg/mL or N-terminal pro–B-type natriuretic peptide ≥1,600 pg/mL, LVEF ≦40%, MR ≥moderate, tricuspid regurgitation ≥moderate, stroke, myocardial infarction, vascular complications, acute kidney injury, bleeding, new pacemaker implantation, and paravalvular leak ≥moderate. The variables included in the IPTW analyses were following: age, sex, body mass index, body surface area, NYHA functional class III or IV, hypertension, dyslipidemia, diabetes mellitus, chronic kidney disease, previous stroke, chronic obstructive pulmonary disease, peripheral artery disease, liver disease, coronary artery disease, previous coronary artery bypass grafting, atrial fibrillation, permanent pacemaker, clinical frailty score, beta-blockers, renin-angiotensin system inhibitors, nontransfemoral approach, local anesthesia, hemoglobin, estimated glomerular filtration rate, albumin <3.5 g/dL, B-type natriuretic peptide ≥400 pg/mL or N-terminal pro–B-type natriuretic peptide ≥1,600 pg/mL, left ventricular ejection fraction, aortic regurgitation ≥moderate, mitral regurgitation ≥moderate, tricuspid regurgitation ≥moderate, balloon expandable valve, stroke, myocardial infarction, vascular complications, acute kidney injury, bleeding, new pacemaker implantation, transcatheter heart valve mean pressure gradient, indexed effective orifice area, and paravalvular leak ≥moderate.

IPTW = inverse probability of treatment weighting; LBBB = left bundle branch block.

**Figure 2:**
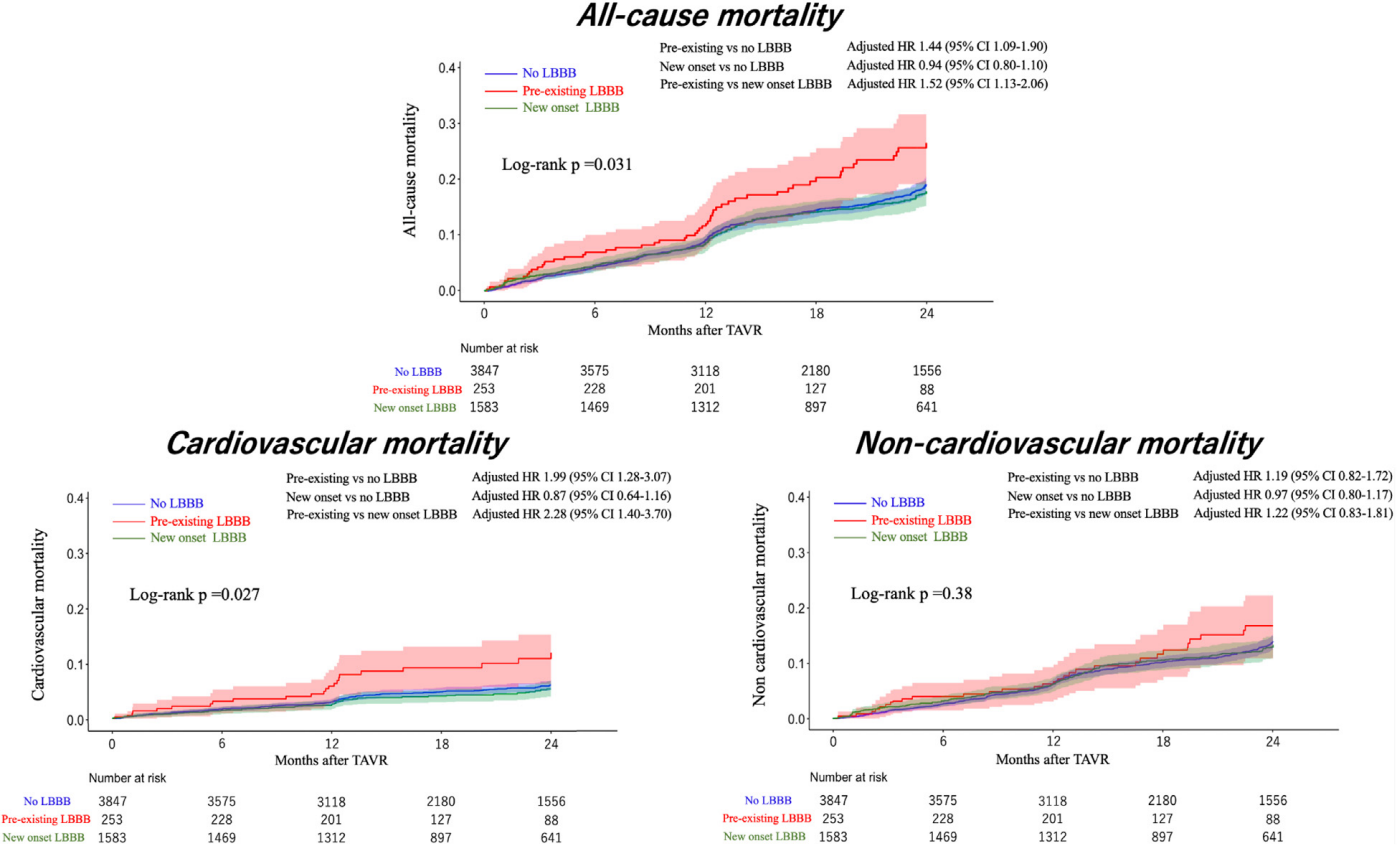
All-Cause, Cardiovascular, and Noncardiovascular Mortality in the Weighted Cohort All-cause and cardiovascular mortality of patients with pre-existing, new onset, and no left bundle branch block (LBBB) in overall and weighted cohorts.

IPTW analyses were performed. The maximum absolute value of the standardized mean difference was <0.1 in all examined covariates in the weighted cohort (Supplemental Table 1). The number of included participants per group is shown Table 2. The comparison of participants included and excluded from IPTW analyses is shown in Supplemental Table 6. Kaplan-Meier curves of all-cause, cardiovascular, and noncardiovascular mortality for the 3 groups are shown in Figure 2. IPTW analyses showed that patients with pre-existing LBBB were associated with higher all-cause (HR: 1.43, 95% CI: 1.09-1.89; *P* = 0.011) and cardiovascular (HR: 1.74, 95% CI: 1.12-2.72; *P* = 0.013) mortality than those without LBBB and were associated with higher all-cause (HR: 1.53, 95% CI: 1.13-2.06; *P* = 0.0058) and cardiovascular (HR: 2.11, 95% CI: 1.29-3.46; *P* = 0.003) mortality than those with new onset LBBB. New onset LBBB was not associated with all-cause (HR: 0.93.; 95% CI: 0.80-1.10; *P* = 0.44) or cardiovascular mortality (HR: 0.82; 95% CI: 0.62-1.11; *P* = 0.21) (Table 2). In the IPTW analysis, there was no significant difference in noncardiac death between the 3 groups.

### Death from heart failure and sudden cardiac death

**Figure 3:**
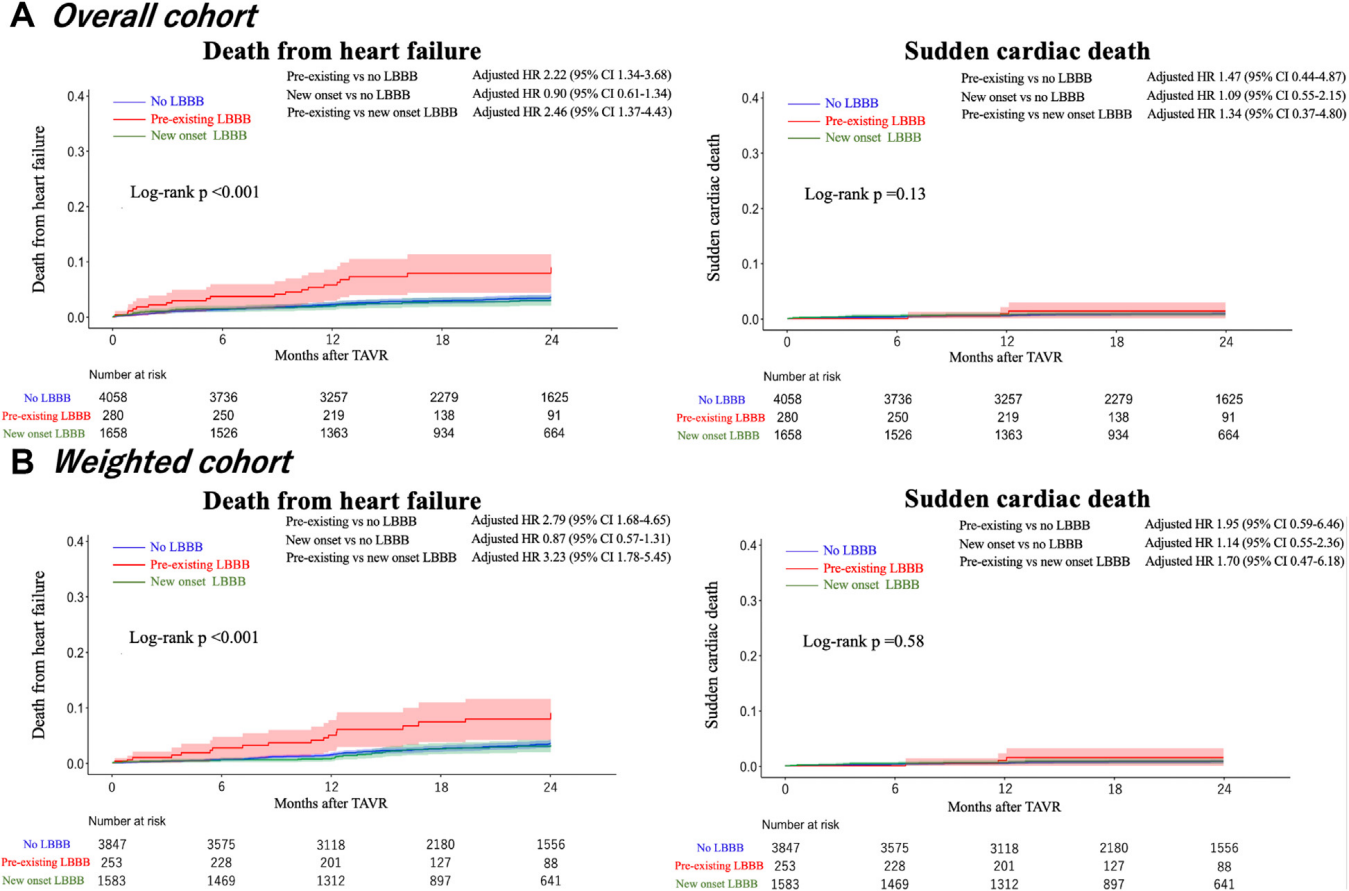
Death From Heart Failure and Sudden Cardiac Death Death from (A) heart failure and (B) sudden cardiac death of patients with pre-existing, new onset, and no left bundle branch block (LBBB) in the unweighted and weighted cohorts.

The causes of cardiovascular death in each group are shown in Central Illustration. Heart failure and SCD were the 2 leading causes of death in all groups. The Kaplan-Meier curves for heart failure death and SCD before and after IPTW are shown in Figure 3. During the follow-up period, 173 patients died of heart failure, 43 of whom died of SCD. The number of included participants per group in the IPTW analyses is shown in Table 3. Heart failure death was significantly more common in patients with pre-existing LBBB (log-rank *P <* 0.001). Occurrence of SCD was not significantly different among the 3 groups (log-rank *P =* 0.13). In multivariable Cox analysis of heart failure death, patients with pre-existing LBBB were associated with a higher risk of heart failure death (adjusted HR: 2.22; 95% CI: 1.34-3.68; *P* = 0.0018) than those without LBBB and an even higher risk of heart failure death (adjusted HR: 2.46; 95% CI: 1.37-4.43; *P* = 0.0025) than those with new onset LBBB (Table 3). The IPTW analysis likewise showed that patients with pre-existing LBBB not only had more heart failure deaths than those without LBBB (adjusted HR: 2.79; 95% CI: 1.68-4.65; *P* < 0.001), but also had more heart failure deaths than those with new onset LBBB (adjusted HR: 3.23; 95% CI: 1.78-5.85; *P* < 0.001). Neither multivariable nor IPTW analysis showed any significant difference in SCD between the 3 groups (Table 3). The full univariable and multivariable model results are shown in Supplemental Tables 7 and 8.

**Table 3:** Analyses of Death From Heart Failure and Sudden Cardiac Death

	Number of Events/N	Vs No LBBB		Vs New Onset LBBB	
		HR (95% CI)	*P* Value	HR (95% CI)	*P* Value
Unadjusted					
Death from heart failure					
No LBBB	676/4,058	Reference		1.11 (0.96-1.29)	0.13
Pre-existing LBBB	64/280	1.44 (1.12-1.87)	0.004	1.61 (1.22-2.12)	0.001
New onset LBBB	248/1,658	0.89 (0.77-1.03)	0.13	Reference	
Sudden cardiac death					
No LBBB	220/4,058	Reference		1.18 (0.91-1.54)	0.19
Pre-existing LBBB	25/280	1.72 (1.14-2.60)	0.01	2.04 (1.29-3.21)	0.001
New onset LBBB	76/1,658	0.84 (0.65-1.09)	0.19	Reference	
Multivariable Cox regression analyses					
Death from heart failure					
No LBBB	676/4,058	Reference		1.02 (0.87-1.19)	0.75
Pre-existing LBBB	64/280	1.39 (1.06-1.82)	0.015	1.43 (1.07-1.91)	0.016
New onset LBBB	248/1,658	0.97 (0.83-1.13)	0.75	Reference	
Sudden cardiac death					
No LBBB	220/4,058	Reference		1.13 (0.85-1.50)	0.39
Pre-existing LBBB	25/280	1.62 (1.05-2.50)	0.028	1.84 (1.14-2.98)	0.012
New onset LBBB	76/1,658	0.88 (0.66-1.17)	0.39	Reference	
IPTW analyses					
Death from heart failure					
No LBBB	148/5,685	Reference		1.15 (0.76-1.74)	0.49
Pre-existing LBBB	103/1,480	2.79 (1.68-4.65)	0.001	3.23 (1.78-5.85)	0.001
New onset LBBB	125/5,567	0.86 (0.57-1.30)	0.49	Reference	
Sudden cardiac death					
No LBBB	37/5,685	Reference		0.87 (0.42-1.80)	0.71
Pre-existing LBBB	18.4/1,480	1.95 (0.59-6.46)	0.27	1.70 (0.47-6.18)	0.41
New onset LBBB	41.5/5,567	1.14 (0.55-2.36)	0.71	Reference	

The variables included in the multivariate Cox regression analyses for death from heart failure were following: age, sex, NYHA functional class III or IV, diabetes mellitus, chronic kidney disease, atrial fibrillation, previous stroke, peripheral artery disease, previous coronary artery bypass grafting, permanent pacemaker, B-type natriuretic peptide ≥400 pg/mL or N-terminal pro–B-type natriuretic peptide ≥1,600 pg/mL, left ventricular ejection fraction ≤40%, tricuspid regurgitation ≥moderate, myocardial infarction, acute kidney injury, bleeding, and paravalvular leak ≥moderate. The variables included in the multivariate Cox regression analyses for sudden cardiac death were following: NYHA functional class III or IV, albumin <3.5 g/dL, and bleeding. The variables included in the IPTW analyses were following: age, sex, body mass index, body surface area, NYHA functional class III or IV, hypertension, dyslipidemia, diabetes mellitus, chronic kidney disease, previous stroke, chronic obstructive pulmonary disease, peripheral artery disease, liver disease, coronary artery disease, previous coronary artery bypass grafting, atrial fibrillation, permanent pacemaker, clinical frailty score, beta-blockers, renin-angiotensin system inhibitors, nontransfemoral approach, local anesthesia, hemoglobin, estimated glomerular filtration rate, albumin <3.5 g/dL, B-type natriuretic peptide ≥400 pg/mL or N-terminal pro–B-type natriuretic peptide ≥1,600 pg/mL, left ventricular ejection fraction, aortic regurgitation ≥moderate, mitral regurgitation ≥moderate, tricuspid regurgitation ≥moderate, balloon expandable valve, stroke, myocardial infarction, vascular complications, acute kidney injury, bleeding, new pacemaker implantation, transcatheter heart valve mean pressure gradient, indexed effective orifice area, and paravalvular leak ≥moderate.

Abbreviations as in Table 2.

### Subgroup analyses

Subgroup analyses for all-cause mortality are shown in Supplemental Figure 3. There were no significant interactions between pre-existing LBBB and prespecified subgroups.

### Sensitivity analyses

Sensitivity analyses showed a curved surface representing the fully adjusted HRs for pre-existing LBBB using new onset LBBB as reference (Supplemental Figure 4). The prevalence of an unmeasured confounder in the new onset LBBB group (P_C0_) was set at 0.5. The prevalence of the unmeasured confounder in the pre-existing LBBB group (P_C1_) varied between 0.0 and 1.0 on the x-axis. The strength of the confounder-outcome association (HR_CD_) varied between 1.0 and 5.5 on the z-axis. In all-cause and cardiovascular mortality, all adjusted HRs were >1 in different settings.

### Post hoc analyses

The results of the post hoc analyses are shown in Supplemental Table 9. The results were similar to those of the main analyses.

## Discussion

The impact of pre-existing LBBB on patients undergoing TAVR remains unknown. The main findings of our study showed that pre-existing LBBB was associated with poor clinical outcomes after TAVR. Furthermore, patients with pre-existing LBBB had worse prognoses than those with new onset LBBB. To the best of our knowledge, the present study is the first to identify the association of pre-existing LBBB and a high risk of mortality after TAVR.

Most reports of LBBB in patients who underwent TAVR pertain to postprocedural new onset LBBB. The prognostic impact of new onset LBBB is controversial.^5^, ^6^, ^7^ However, these reports exclude patients with pre-existing LBBB. As such, few studies are available on the impact of pre-existing LBBB on clinical outcomes after TAVR. Fischer et al^8^ reported that pre-existing LBBB is a risk for early PPI after TAVR, but not for late PPI, and, furthermore, has no significant effect on mortality after TAVR. However, in our study, pre-existing LBBB was not a risk for early PPI. However, the patients with pre-existing LBBB had a higher rate of prior pacemaker implantation, precluding post-TAVR pacemaker implantation. In terms of complications, bleeding and pacemaker implantation were more common in patients with new onset LBBB. However, complications were similar for patients with pre-existing LBBB and no LBBB.

In our study, 4.6% of patients undergoing TAVR had LBBB. This percentage is greater than that of LBBB in the general population.^27^ However, the percentage of pre-existing LBBB patients undergoing TAVR reported so far is around 10%,^8^ and the incidence in our study is somewhat lower than that before. Also, although LBBB increases with age, valvular heart disease, coronary heart disease, cardiomyopathies, and myocarditis can be background factors for LBBB. Because patients with severe AS are older and therefore at higher risk than the general population, the rate of LBBB is also likely to be higher than that in the general population. In our study, patients with LBBB were more symptomatic and had worse renal functions, higher BNP, larger left ventricular diameters, lower LVEF, and more severe MR. LBBB causes dyssynchronous left ventricular activation and left ventricular remodeling, resulting in left ventricular dilatation, low LVEF, and MR. Worse outcomes in heart failure patients with LBBB may be expected due to dyssynchronous left ventricular activation. In fact, LBBB is associated with increased all-cause mortality not only in patients with heart failure but also in those with ischemic heart disease, cardiomyopathy, and even in the general population.^27^ Based on the findings of prior studies, potential mechanisms of our results may include progression to high-degree AVB, ventricular arrhythmia, or worsening heart failure. However, in our study, many deaths were due to heart failure, not SCD.

In our study, patients with pre-existing LBBB had worse prognoses than those with new onset LBBB. We also performed sensitivity analyses on the HR of pre-existing LBBB using new onset LBBB as a reference, but even under many assumptions, the pre-existing LBBB group had a worse prognosis than the new onset LBBB group. There have been mixed reports on the possible risk of new onset LBBB with respect to all-cause death, cardiovascular death, heart failure hospitalization, and new pacemaker implantation.^28^ In our study, new onset LBBB was determined only by post-TAVR electrocardiography before or at discharge, and it is possible that some of the patients diagnosed with new onset LBBB may have improved during follow-up. Therefore, it should be noted that the present results are the result of a mixture of transient and persist postprocedural LBBB. It has been reported that transient LBBB is not associated with prognosis,^29^ and our study may have included more transient LBBB than previous studies. In addition, new onset LBBB is caused by mechanical compression of the conduction system by a prosthetic valve, but pre-existing LBBB is due to a failure of one's own conduction system, which may be hiding cardiomyopathy or other problems that were not fully understood in our study, which may have led to our results. It has been reported that bundle branch block is more common in patients with amyloidosis,^30^ and since our study excluded patients with RBBB, it is possible that there were more patients with amyloidosis in the pre-existing group.

There is no available evidence for treatment of pre-existing or new onset LBBB after TAVR. Thus, further research is needed to determine the timing of device implantation and which devices, including cardiac resynchronization therapy, are best. Because the mortality rate is higher in patients with pre-existing LBBB, it may be important to know whether patients with LBBB after TAVR have pre-existing LBBB or new onset LBBB. Although perioperative risks are not high, patients with pre-existing LBBB should be carefully monitored after TAVR. However, currently, there are no reports other than our study showing similar results. Further investigation is required.

### Study limitations

First, as shown in Supplemental Table 2, patients not included in the study may have a poor prognosis, leading to selection bias. This may be related to the fact that patients with RBBB have a poor prognosis,^3^^,^^4^ and those without electrocardiography recordings are in poor condition. Second, no detailed electrocardiographic information, such as PR interval and QRS duration, is available. QRS duration is important, as it has been reported to be associated with prognosis.^31^ In addition to new onset LBBB, there are also reports that first degree of atrioventricular block is a risk for PPI.^32^ Third, the indication of PPI during follow-up was not collected although the frequency of PPI during follow-up is important. Fourth, follow-up medication and echocardiographic data are scarce. There are reports that beta-blockers and renin-angiotensin inhibitors were associated with lower mortality in patients who underwent TAVR.^33^^,^^34^ The optimal medical therapy may be important because patients with pre-existing LBBB have lower ejection fraction than those with no LBBB. Fifth, there is a lack of procedural and computed tomography data. The length of the membrane septum and the depth of valve implantation are relevant for new onset LBBB and permanent pacemaker implantation.^35^^,^^36^ Sixth, although the multivariate Cox regression and IPTW analyses were performed, the baselines of each group differed greatly. This study was an observational study and could only be adjusted with measured covariates. Therefore, the selection bias was inevitable and baseline characteristics were not completely aligned. Finally, although 3-group comparisons were used in this study, multiple comparisons are prone to statistical errors. Therefore, more meticulous studies that consider these limitations should be our future targets.

## Conclusions

Pre-existing LBBB was independently associated with poor outcomes, reflecting increased risk of cardiovascular mortality after TAVR. Patients with pre-existing LBBB should be carefully monitored after TAVR. Further investigation will be required to corroborate our findings.

Perspectives **COMPETENCY IN MEDICAL KNOWLEDGE:** Currently, few reports are available on pre-existing LBBB in patients undergoing TAVR.

**TRANSLATIONAL OUTLOOK:** Using data from the Japanese multicenter registry, this study aimed to investigate the association between pre-existing or new onset LBBB and clinical outcomes after TAVR. Pre-existing LBBB was independently associated with poor clinical outcomes, reflecting an increased risk of cardiovascular mortality after TAVR. Patients with pre-existing LBBB should be carefully monitored after TAVR.

## Funding Support and Author Disclosures

The OCEAN-TAVI registry is supported by Edwards Lifesciences, Medtronic, Boston Scientific, Abbott Medical, and Daiichi-Sankyo. Dr Izumo have served as a screening proctor for Edwards Lifesciences and a clinical proctor for Abbott Medical. Drs Shimizu and Takagi served as clinical proctors for Edwards Lifesciences. Drs Yashima, Ohno, and Asami served as clinical proctors for Medtronic. Drs Naganuma, Mizutani, Tada, Ueno, and Watanabe served as clinical proctors for Edwards Lifesciences and Medtronic. Drs Shirai, Yamamoto, and Hayashida served as clinical proctors for Edwards Lifesciences, Abbott Medical, and Medtronic. All other authors have reported that they have no relationships relevant to the contents of this paper to disclose.
