# Supplementary material for: Pre-Existing Left Bundle Branch Block and Clinical Outcomes After Transcatheter Aortic Valve Replacement
Source: JACC Asia. 2024 Jan 9;4(4):306–19. doi: 10.1016/j.jacasi.2023.11.007 (PMC11035950; doi:10.1016/j.jacasi.2023.11.007)
Supplement: Supplemental Tables 1-9 and Supplemental Figures 1-4 [file mmc1.docx]

**Pre-Existing Left Bundle Branch Block and Clinical Outcomes After Transcatheter Aortic Valve Replacement**

Tetsuya Saito, MD, Taku Inohara, MD, Hikaru Tsuruta, MD, Fumiaki Yashima, MD, Hideyuki Shimizu, MD, Keiichi Fukuda, MD, Yohei Ohno, MD, Hidetaka Nishina, MD, Masaki Izumo, MD, Masahiko Asami, MD, Toru Naganuma, MD, Kazuki Mizutani, MD, Masahiro Yamawaki, MD, Norio Tada, MD, Futoshi Yamanaka, MD, Shinichi Shirai, MD, Masahiko Noguchi, MD, Hiroshi Ueno, MD, Kensuke Takagi, MD, Yusuke Watanabe, MD, Masanori Yamamoto, MD, Kentaro Hayashida, MD, on behalf of the OCEAN-TAVI investigators

**Supplemental Table 1. Absolute value of the maximum standardized mean difference before and after inverse probability treatment weighting.**

|  | Absolute value of maximum standardized mean difference | |
| --- | --- | --- |
|  | Unweighted | Weighted |
| Age, yrs | 0.18 | 0.093 |
| Male | 0.11 | 0.0089 |
| Body mass index, kg/m^2^ | 0.14 | 0.013 |
| Body surface area, m^2^ | 0.17 | 0.027 |
| NYHA 3 or 4 | 0.16 | 0.034 |
| Hypertension | 0.017 | 0.014 |
| Dyslipidemia | 0.035 | 0.006 |
| Diabetes mellitus | 0.011 | 0.025 |
| Chronic kidney disease | 0.074 | 0.0015 |
| Previous stroke | 0.012 | 0.0064 |
| COPD | 0.029 | 0.0013 |
| Peripheral artery disease | 0.023 | 0.023 |
| Liver disease | 0.0068 | 0.0054 |
| Coronary artery disease | 0.029 | 0.01 |
| Previous CABG | 0.021 | 0.0089 |
| Atrial fibrillation | 0.029 | 0.048 |
| Permanent pacemaker | 0.075 | 0.012 |
| Clinical frailty scale | 0.096 | 0.069 |
| Beta blockers | 0.023 | 0.0089 |
| RAS inhibitors | 0.029 | 0.0082 |
| STS risk score, % | 0.33 | 0.081 |
| Hemoglobin, g/dl | 0.032 | 0.053 |
| eGFR, ml/min/1.73m^2^ | 0.11 | 0.09 |
| Albumin < 3.5 g/dl | 0.022 | 0.011 |
| BNP ≧ 400 pg/ml or NT-Pro BNP ≧ 1600 pg/ml | 0.19 | 0.001 |
| LVEF, % | 0.94 | 0.083 |
| Aortic regurgitation ≥moderate | 0.031 | 0.0067 |
| Mitral regurgitation ≥moderate | 0.071 | 0.0032 |
| Tricuspid regurgitation ≥moderate | 0.022 | 0.046 |
| Non-transfemoral approach | 0.037 | 0.0059 |
| Local anesthesia | 0.016 | 0.012 |
| Balloon expandable valve | 0.086 | 0.0082 |
| Stroke | 0.0058 | 0.0015 |
| Myocardial infarction | 0.0055 | 0.0021 |
| Vascular complications | 0.0099 | 0.0073 |
| AKI | 0.0229 | 0.0075 |
| Bleeding | 0.03 | 0.0044 |
| New pacemaker implantation | 0.025 | 0.0015 |
| THV mean pressure gradient | 0.1 | 0.008 |
| Indexed EOA | 0.13 | 0.017 |
| PVL ≥moderate | 0.0016 | 0.0036 |

AKI indicates acute kidney injury; AR, aortic regurgitation; BNP, brain natriuretic peptide; CABG, coronary artery bypass grafting; COPD, chronic obstructive pulmonary disease; EOA, effective orifice area; LVEF, left ventricular ejection fraction; MR, mitral regurgitation; NYHA, New York Heart Association; PVL, paravalvular leak; STS, Society of Thoracic Surgeons; THV, transcatheter heart valve; and TR, tricuspid regurgitation.

**Supplemental Table 2. Comparison of patients who were included and excluded from the study**

|  | Included patients  n=5996 | Excluded patients  N=1397 | P value |
| --- | --- | --- | --- |
| Pre-procedural clinical data | | | |
| Age, yrs | 85 (81-88) | 85 (82-88) | <0.001 |
| Male | 1811 (30.2) | 552 (39.5) | <0.001 |
| Body mass index, kg/m^2^ | 22.1 (19.6-24.5) | 22.2 (19.7-24.6) | 0.23 |
| Body surface area, m^2^ | 1.40 (1.30-1.55) | 1.44 (1.30-1.60) | <0.001 |
| NYHA 3 or 4 | 2426 (40.5) | 607 (43.5) | 0.041 |
| Hypertension | 5014 (83.6) | 1163 (83.2) | 0.73 |
| Dyslipidemia | 3336 (55.6) | 760 (54.4) | 0.4 |
| Diabetes mellitus | 1621 (27.0) | 379 (27.1) | 0.93 |
| Chronic kidney disease | 4150 (69.2) | 1028 (73.6) | 0.001 |
| Previous stroke | 670 (11.2) | 162 (11.6) | 0.65 |
| COPD | 568 (9.5) | 138 (9.9) | 0.64 |
| Peripheral artery disease | 658 (11.0) | 169 (12.1) | 0.23 |
| Coronary artery disease | 1954 (32.6) | 491 (35.1) | 0.067 |
| Previous CABG | 266 (4.4) | 67 (4.8) | 0.55 |
| Atrial fibrillation | 1236 (20.6) | 338 (24.2) | 0.003 |
| Permanent pacemaker | 176 (2.6) | 236 (16.9) | <0.001 |
| Clinical frailty scale | 4 (3-4) | 4 (3-4) | 0.29 |
| Beta blockers | 1988 (33.2) | 473 (33.9) | 0.64 |
| RAS inhibitors | 3132 (52.2) | 771 (55.2) | 0.044 |
| STS risk score, % | 6.18 (4.21-9.19) | 6.28 (4.44-9.23) | 0.094 |
| Non-transfemoral approach | 568 (9.5) | 197 (14.1) | 0.002 |
| Local anesthesia | 2196 (36.6) | 467 (33.4) | 0.025 |
| Hemoglobin, g/dl | 11.4 (10.2-12.5) | 11.3 (10.1-12.5) | 0.11 |
| eGFR, ml/min/1.73m^2^ | 50.8 (38.4-63.6) | 47.5 (36.0-60.5) | <0.001 |
| Albumin, g/dl | 3.80 (3.50-4.10) | 3.80 (3.40-4.10) | 0.56 |
| BNP, pg/ml | 235 (101-518) | 255 (120-556) | 0.021 |
| BNP ≥400 pg/ml or NT-Pro BNP ≥1600 pg/ml | 2081 (35.2) | 530 (38.5) | 0.023 |
| Pre-procedural echocardiographic data | | | |
| Aortic valve area, cm^2^ | 0.63 (0.50-0.76) | 0.64 (0.51-0.77) | 0.047 |
| Peak velocity, m/s | 4.50 (4.04-5.08) | 4.40 (4.00-4.96) | <0.001 |
| Mean pressure gradient, mmHg | 47.0 (37.0-61.0) | 45.0 (35.0-58.7) | 0.001 |
| LV end-diastolic dimension, mm | 43.3 (39.7-47.8) | 44.0 (40.0-49.0) | 0.033 |
| LV end-systolic dimension, mm | 28.0 (24.4-32.5) | 28.0 (24.0-33.0) | 0.58 |
| LVEF, % | 62.8 (54.0-68.1) | 63.0 (54.0-69.0) | 0.088 |
| Aortic regurgitation ≥moderate | 618 (10.3) | 168 (2.0) | 0.06 |
| Mitral regurgitation ≥moderate | 671 (11.2) | 197 (14.1) | 0.002 |
| Tricuspid regurgitation ≥moderate | 498 (8.3) | 167 (12.0) | <0.001 |
| Non-transfemoral approach | 25 (8.9) | 111 (6.7) | <0.001 |
| Local anesthesia | 2196 (36.6) | 467 (33.4) | 0.025 |
| Balloon expandable valves | 4571 (76.2) | 1044 (74.7) | 0.23 |
| Post-procedural outcomes and complications | | | |
| 30-day mortality | 75 (1.3) | 27 (1.9) | 0.049 |
| Stroke | 144 (2.4) | 25 (1.8) | 0.16 |
| Myocardial infarction | 34 (0.6) | 8 (0.6) | 0.98 |
| Vascular complications | 445 (9.0) | 90 (8.2) | 0.4 |
| AKI | 479 (8.0) | 148 (10.6) | 0.02 |
| Bleeding | 832 (16.7) | 201 (17.9) | 0.31 |
| New pacemaker | 223 (3.7) | 360 (25.8) | <0.001 |
| Indexed EOA, cm^2^/m^2^ | 1.07 (0.90-1.29) | 1.08 (0.91-1.25) | 0.87 |
| THV mean pressure gradient, mmHg | 10.2 (7.9-13.8) | 10.1 (7.7-13.6) | 0.71 |
| PVL ≥moderate | 118 (2.0) | 30 (2.2) | 0.64 |

AKI indicates acute kidney injury; AR, aortic regurgitation; BNP, brain natriuretic peptide; CABG, coronary artery bypass grafting; COPD, chronic obstructive pulmonary disease; EOA, effective orifice area; LVEF, left ventricular ejection fraction; MR, mitral regurgitation; NYHA, New York Heart Association; PVL, paravalvular leak; STS, Society of Thoracic Surgeons; THV, transcatheter heart valve; and TR, tricuspid regurgitation.

**Supplemental Table 3. Univariable and multivariable Cox regression analysis of 2-year all-cause mortality.**

|  | Univariable analysis | | Multivariable analysis | |
| --- | --- | --- | --- | --- |
|  | HR (95% CI) | P value | HR (95% CI) | P value |
| Pre-existing LBBB (vs no LBBB) | 1.44 (1.12-1.87) | 0.0049 | 1.39 (1.06-1.82) | 0.015 |
| New onset LBBB (vs no LBBB) | 0.89 (0.77-1.03) | 0.13 | 0.97 (0.83-1.13) | 0.75 |
| Pre-existing LBBB (vs new onset LBBB) | 1.61 (1.22-2.12) | <0.001 | 1.43 (1.07-1.91) | 0.016 |
| Age (per increase) | 1.02 (1.01-1.03) | <0.001 | 1.00 (0.99-1.01) | 0.48 |
| Male | 1.40 (1.23-1.59) | <0.001 | 1.99 (1.67-2.38) | <0.001 |
| Body surface index (per 1m^2^ increase) | 0.46 (0.32-0.66) | <0.001 | 0.30 (0.18-0.51) | <0.001 |
| NYHA 3 or 4 | 1.82 (1.61-2.06) | <0.001 | 1.24 (1.08-1.43) | 0.0019 |
| Dyslipidemia | 0.64 (0.57-0.72) | <0.001 | 0.76 (0.66-0.87) | <0.001 |
| Diabetes mellitus | 1.24 (1.09-1.42) | <0.001 | 1.28 (1.11-1.49) | <0.001 |
| Chronic kidney disease | 1.29 (1.09-1.48) | <0.001 | 1.01 (0.86-1.17) | 0.94 |
| Atrial fibrillation | 1.51 (1.31-1.72) | <0.001 | 1.18 (1.01-1.37) | 0.034 |
| COPD | 1.55 (1.30-1.85) | <0.001 | 1.30 (1.07-1.57) | 0.006 |
| Peripheral artery disease | 1.83 (1.56-2.15) | <0.001 | 1.31 (1.10-1.57) | 0.0026 |
| Previous CABG | 1.41 (1.09-1.83) | 0.008 | 1.24 (0.94-1.64) | 0.12 |
| Previous pacemaker | 1.52 (1.13-2.04) | 0.0046 | 1.15 (0.84-1.58) | 0.36 |
| Previous stroke | 1.26 (1.05-1.51) | 0.011 | 1.01 (0.83-1.23) | 0.91 |
| Clinical frail score (per 1 group increase) | 1.32 (1.26-1.38) | <0.001 | 1.15 (1.09-1.21) | <0.001 |
| Non-transfemoral approach | 1.70 (1.43-2.02) | <0.001 | 1.12 (0.92-1.38) | 0.24 |
| Local anesthesia | 0.83 (0.73-0.95) | 0.007 | 0.98 (0.84-1.13) | 0.79 |
| Hemoglobin (per 1g/dl increase) | 0.82 (0.78-0.85) | <0.001 | 0.91 (0.87-0.95) | <0.001 |
| Albumin <3.5g/dl | 2.72 (2.40-3.08) | <0.001 | 1.66 (1.43-1.92) | <0.001 |
| BNP ≧400 pg/ml or NT-Pro BNP ≧1600 pg/ml | 1.85 (1.64-2.10) | <0.001 | 1.18 (1.02-1.37) | 0.02 |
| LVEF ≦40% | 1.51 (1.25-1.83) | <0.001 | 0.84 (0.68-1.06) | 0.14 |
| MR ≧moderate | 1.25 (1.04-1.50) | 0.014 | 0.85 (0.69-1.04) | 0.13 |
| AR ≧moderate | 0.96 (0.78-1.18) | 0.71 |  |  |
| TR ≧moderate | 1.53 (1.27-1.85) | <0.001 | 1.22 (0.98-1.51) | 0.065 |
| Stroke | 2.26 (1.68-2.37) | 0.004 | 1.54 (1.11-2.16) | 0.01 |
| Myocardial infarction | 2.10 (1.16-3.81) | 0.013 | 1.03 (0.51-2.10) | 0.92 |
| Vascular complications | 1.58 (1.29-1.92) | <0.001 | 1.05 (0.83-1.33) | 0.66 |
| AKI | 2.95 (2.51-3.81) | <0.001 | 1.74 (1.44-2.10) | <0.001 |
| Bleeding | 2.03 (1.76-2.35) | <0.001 | 1.42 (1.20-1.69) | <0.001 |
| New pacemaker implantation | 1.17 (0.90-1.51) | 0.21 | 1.03 (0.74-1.42) | 0.85 |
| PVL ≧moderate | 1.67 (1.17-2.37) | 0.004 | 1.27 (0.87-1.86) | 0.2 |

AKI indicates acute kidney injury; AR, aortic regurgitation; BNP, brain natriuretic peptide; CABG, coronary artery bypass grafting; COPD, chronic obstructive pulmonary disease; LVEF, left ventricular ejection fraction; MR, mitral regurgitation; NYHA, New York Heart Association; PVL, paravalvular leak; and TR, tricuspid regurgitation.

**Supplemental Table 4. Univariable and multivariable Cox regression analysis of 2-year cardiovascular mortality.**

|  | Univariable analysis | | Multivariable analysis | |
| --- | --- | --- | --- | --- |
|  | HR (95% CI) | P value | HR (95% CI) | P value |
| Pre-existing LBBB (vs no LBBB) | 1.72 (1.14-2.60) | 0.01 | 1.60 (1.04-2.48) | 0.031 |
| New onset LBBB (vs no LBBB) | 0.84 (0.65-1.09) | 0.19 | 0.88 (0.66-1.17) | 0.39 |
| Pre-existing LBBB (vs new onset LBBB) | 2.04 (1.29-3.21) | 0.0019 | 1.81 (1.12-2.93) | 0.014 |
| Age (per 1 yr increase) | 1.01 (0.99-1.04) | 0.11 | 0.98 (0.96-1.01) | 0.33 |
| Male | 1.21 (0.96-1.52) | 0.01 | 1.78 (1.29-2.45) | <0.001 |
| Body surface index (per 1m^2^ increase) | 0.31 (0.16-0.61) | <0.001 | 0.18 (0.07-0.45) | <0.001 |
| NYHA 3 or 4 | 2.34 (1.87-2.93) | <0.001 | 1.58 (1.22-2.04) | <0.001 |
| Dyslipidemia | 0.79 (0.63-0.98) | 0.035 | 0.92 (0.72-1.17) | 0.53 |
| Diabetes mellitus | 1.36 (1.08-1.72) | 0.0088 | 1.38 (1.07-1.79) | 0.012 |
| Chronic kidney disease | 1.53 (1.18-1.99) | 0.0012 | 1.18 (0.88-1.57) | 0.25 |
| Atrial fibrillation | 1.86 (1.47-2.35) | <0.001 | 1.37 (1.05-1.78) | 0.017 |
| COPD | 1.26 (0.89-1.77) | 0.17 | 1.10 (0.77-1.59) | 0.57 |
| Peripheral artery disease | 1.70 (1.26-2.28) | <0.001 | 1.11 (0.79-1.54) | 0.52 |
| Previous CABG | 1.86 (1.23-2.80) | 0.0028 | 1.60 (1.03-2.47) | 0.033 |
| Previous pacemaker | 2.21 (1.40-3.47) | <0.001 | 1.53 (0.93-2.50) | 0.087 |
| Previous stroke | 1.07 (0.76-1.51) | 0.66 | 1.19 (9,57-1.21) | 0.35 |
| Clinical frail score (per 1 group increase) | 1.30 (1.20-1.41) | <0.001 | 1.09 (0.99-1.20) | 0.075 |
| Non-transfemoral approach | 1.81 (1.34-2.45) | <0.001 | 1.10 (0.77-1.57) | 0.58 |
| Local anesthesia | 0.73 (0.57-0.93) | 0.011 | 0.99 (0.76-1.29) | 0.95 |
| Hemoglobin (per 1g/dl increase) | 0.84 (0.79-0.91) | <0.001 | 0.97 (0.90-1.06) | 0.57 |
| Albumin <3.5g/dl | 2.25 (1.80-2.82) | <0.001 | 1.33 (1.02-1.73) | 0.035 |
| BNP ≧400 pg/ml or NT-Pro BNP ≧1600 pg/ml | 2.16 (1.73-2.70) | <0.001 | 1.24 (0.95-1.61) | 0.11 |
| LVEF ≦40% | 1.89 (1.39-2.58) | <0.001 | 0.97 (0.67-1.39) | 0.87 |
| MR ≧moderate | 1.41 (1.03-1.93) | 0.027 | 0.77 (0.54-1.10) | 0.16 |
| AR ≧moderate | 0.87 (0.59-1.27) | 0.48 |  |  |
| TR ≧moderate | 2.31 (1.72-3.11) | <0.001 | 1.90 (1.35-2.67) | <0.001 |
| Stroke | 2.43 (1.47-4.00) | <0.001 | 1.43 (0.80-2.56) | 0.21 |
| Myocardial infarction | 5.76 (2.97-11.1) | <0.001 | 2.67 (1.23-5.77) | 0.012 |
| Vascular complications | 1.65 (1.17-2.34) | 0.004 | 0.85 (0.56-1.29) | 0.46 |
| AKI | 3.56 (2.72-4.67) | <0.001 | 2.05 (1.49-2.83) | <0.001 |
| Bleeding | 2.67 (2.09-3.41) | <0.001 | 1.91 (1.43-2.55) | <0.001 |
| New pacemaker implantation | 1.16 (0.68-1.91) | 0.57 | 0.99 (0.55-1.78) | 0.97 |
| PVL ≧moderate | 2.16 (1.21-3.85) | 0.0088 | 1.59 (0.87-2.92) | 0.12 |

AKI indicates acute kidney injury; AR, aortic regurgitation; BNP, brain natriuretic peptide; CABG, coronary artery bypass grafting; COPD, chronic obstructive pulmonary disease; LVEF, left ventricular ejection fraction; MR, mitral regurgitation; NYHA, New York Heart Association; PVL, paravalvular leak; and TR, tricuspid regurgitation.

**Supplemental Table 5. Univariable and multivariable Cox regression analysis of 2-year non-cardiovascular mortality.**

|  | Univariable analysis | | Multivariable analysis | |
| --- | --- | --- | --- | --- |
|  | HR (95% CI) | P value | HR (95% CI) | P value |
| Pre-existing LBBB (vs no LBBB) | 1.31 (0.94-1.81) | 0.11 | 1.26 (0.89-1.78) | 0.19 |
| New onset LBBB (vs no LBBB) | 0.91 (0.77-1.09) | 0.34 | 1.01 (0.84-1.21) | 0.89 |
| Pre-existing LBBB (vs new onset LBBB) | 1.46 (1.00-2.08) | 0.046 | 1.24 (0.86-1.79) | 0.24 |
| Age (per 1 yr increase) | 1.03 (1.01-1.05) | <0.001 | 1.01 (0.99-1.02) | 0.11 |
| Male | 1.50 (1.28-1.75) | <0.001 | 2.08 (1.69-2.57) | <0.001 |
| Body surface index (per 1m^2^ increase) | 0.55 (0.35-0.86) | 0.009 | 0.39 (0.21-0.74) | 0.0039 |
| NYHA 3 or 4 | 1.59 (1.37-1.85) | <0.001 | 1.12 (0.94-1.33) | 0.17 |
| Dyslipidemia | 0.59 (0.51-0.69) | <0.001 | 0.68 (0.58-0.81) | <0.001 |
| Diabetes mellitus | 1.19 (1.01-1.41) | 0.033 | 1.24 (1.04-1.49) | 0.015 |
| Chronic kidney disease | 1.16 (0.98-1.38) | 0.069 | 0.93 (0.77-1.12) | 0.44 |
| Atrial fibrillation | 1.32 (1.11-1.58) | 0.0016 | 1.08 (0.89-1.30) | 0.42 |
| COPD | 1.65 (1.33-2.04) | <0.001 | 1.39 (1.11-1.73) | 0.0037 |
| Peripheral artery disease | 1.91 (1.56-2.32) | <0.001 | 1.42 (1.15-1.76) | 0.0011 |
| Previous CABG | 1.15 (0.81-1.63) | 0.42 | 1.07 (0.74-1.54) | 0.71 |
| Previous pacemaker | 1.24 (0.83-1.87) | 0.28 | 0.99 (0.65-1.49) | 0.96 |
| Previous stroke | 1.33 (1.07-1.66) | 0.01 | 1.09 (0.86-1.37) | 0.46 |
| Clinical frail score (per 1 group increase) | 1.33 (1.26-1.41) | <0.001 | 1.17 (1.10-1.25) | <0.001 |
| Non-transfemoral approach | 1.65 (1.33-2.05) | <0.001 | 1.14 (0.89-1.47) | 0.27 |
| Local anesthesia | 0.87 (0.74-1.02) | 0.097 | 0.97 (0.82-1.16) | 0.81 |
| Hemoglobin (per 1g/dl increase) | 0.81 (0.76-0.84) | <0.001 | 0.88 (0.84-0.93) | <0.001 |
| Albumin <3.5g/dl | 2.84 (2.44-3.32) | <0.001 | 1.84 (1.55-2.20) | <0.001 |
| BNP ≧400 pg/ml or NT-Pro BNP ≧1600 pg/ml | 1.69 (1.45-1.97) | <0.001 | 1.17 (0.98-1.39) | 0.076 |
| LVEF ≦40% | 1.27 (0.99-1.62) | 0.053 | 0.79 (0.60-1.05) | 0.11 |
| MR ≧moderate | 1.17 (0.93-1.47) | 0.16 | 0.90 (0.69-1.15) | 0.41 |
| AR ≧moderate | 0.96 (0.74-1.24) | 0.78 |  |  |
| TR ≧moderate | 1.20 (0.92-1.56) | 0.16 | 0.95 (0.72-1.26) | 0.75 |
| Stroke | 2.08 (1.42-3.04) | <0.001 | 1.55 (1.03-2.34) | 0.035 |
| Myocardial infarction | 0.30 (0.04-2.16) | 0.23 | 0.19 (0.02-1.37) | 0.099 |
| Vascular complications | 1.40 (1.08-1.81) | 0.01 | 1.15 (0.86-1.53) | 0.33 |
| AKI | 2.62 (2.13-3.23) | <0.001 | 1.58 (1.25-2.00) | 0.001 |
| Bleeding | 1.76 (1.45-2.12) | <0.001 | 1.22 (0.99-1.52) | 0.058 |
| New pacemaker implantation | 1.12 (0.76-1.63) | 0.55 | 1.04 (0.71-1.54) | 0.81 |
| PVL ≧moderate | 1.47 (0.92-2.35) | 0.1 | 1.13 (0.69-1.84) | 0.62 |

AKI indicates acute kidney injury; AR, aortic regurgitation; BNP, brain natriuretic peptide; CABG, coronary artery bypass grafting; COPD, chronic obstructive pulmonary disease; LVEF, left ventricular ejection fraction; MR, mitral regurgitation; NYHA, New York Heart Association; PVL, paravalvular leak; and TR, tricuspid regurgitation.

**Supplemental Table 6. Comparison of participants included and excluded from IPTW analyses**

|  | Patients included in IPTW analyses  n=5155 | Patients excluded from IPTW analyses  n=471 | P value |
| --- | --- | --- | --- |
| Pre-procedural clinical data | | | |
| Age, yrs | 85 (81-88) | 83 (79-87) | <0.001 |
| Male | 1388 (26.9) | 299 (63.5) | <0.001 |
| Body mass index, kg/m^2^ | 22.2 (19.7-24.6) | 21.7 (19.1-24.2) | 0.006 |
| Body surface area, m^2^ | 1.40 (1.30-1.53) | 1.50 (1.40-1.62) | <0.001 |
| NYHA 3 or 4 | 1995 (38.7) | 240 (51.0) | <0.001 |
| Hypertension | 4321 (83.8) | 375 (79.6) | 0.019 |
| Dyslipidemia | 2896 (56.2) | 243 (51.6) | 0.055 |
| Diabetes mellitus | 1368 (26.5) | 139 (29.5) | 0.16 |
| Chronic kidney disease | 3542 (68.7) | 324 (68.8) | 0.97 |
| Previous stroke | 562 (10.9) | 51 (10.8) | 0.96 |
| COPD | 474 (9.2) | 55 (11.7) | 0.077 |
| Peripheral artery disease | 492 (9.5) | 98 (20.8) | <0.001 |
| Coronary artery disease | 1609 (31.2) | 190 (40.3) | <0.001 |
| Previous CABG | 204 (4.0) | 35 (7.4) | <0.001 |
| Atrial fibrillation | 1007 (19.5) | 141 (29.9) | <0.001 |
| Permanent pacemaker | 110 (2.1) | 55 (11.7) | <0.001 |
| Clinical frailty scale | 4 (3-4) | 4 (3-4) | 0.53 |
| Beta blockers | 1699 (33.0) | 160 (34.0) | 0.65 |
| RAS inhibitors | 2744 (53.2) | 202 (42.9) | <0.001 |
| STS risk score, % | 6.01 (4.19-8.90) | 6.48 (4.15-10.3) | 0.11 |
| Non-transfemoral approach | 356 (6.9) | 145 (30.8) | <0.001 |
| Local anesthesia | 1979 (38.4) | 136 (28.9) | <0.001 |
| Hemoglobin, g/dl | 11.4 (10.3-12.5) | 11.3 (10.2-12.5) | 0.29 |
| eGFR, ml/min/1.73m^2^ | 51.0 (39.0-63.6) | 50.0 (36.6-63.9) | 0.21 |
| Albumin, g/dl | 3.80 (3.50-4.10) | 3.70 (3.40-4.10) | 0.076 |
| BNP ≥400 pg/ml or NT-Pro BNP ≥1600 pg/ml | 1828 (35.5) | 254 (53.9) | <0.001 |
| Pre-procedural echocardiographic data | | | |
| Aortic valve area, cm^2^ | 0.63 (0.50-0.76) | 0.66 (0.53-0.77) | 0.008 |
| Peak velocity, m/s | 4.50 (4.06-5.10) | 4.39 (3.90-4.90) | <0.001 |
| Mean pressure gradient, mmHg | 47.1 (37.7-61.3) | 44.0 (35.0-55.4) | <0.001 |
| LV end-diastolic dimension, mm | 43.0 (39.2-47.0) | 46.0 (42.0-50.8) | <0.001 |
| LV end-systolic dimension, mm | 27.6 (24.0-32.0) | 31.5 (27.0-37.5) | <0.001 |
| LVEF, % | 63.0 (55.0-68.5) | 57.0 (46.7-65.0) | <0.001 |
| Aortic regurgitation ≥moderate | 475 (9.2) | 89 (18.9) | <0.001 |
| Mitral regurgitation ≥moderate | 531 (10.3) | 73 (15.5) | <0.001 |
| Tricuspid regurgitation ≥moderate | 431 (8.4) | 39 (8.3) | 0.95 |
| Non-transfemoral approach | 356 (6.9) | 145 (30.8) | <0.001 |
| Local anesthesia | 1979 (38.4) | 136 (28.9) | <0.001 |
| Post-procedural outcomes and complications | | | |
| 30-day mortality | 13 (0.3) | 3 (0.6) | 0.13 |
| Stroke | 112 (2.2) | 8 (1.7) | 0.49 |
| Myocardial infarction | 19 (0.4) | 4 (0.8) | 0.11 |
| Vascular complications | 367 (7.1) | 32 (6.8) | 0.79 |
| AKI | 333 (6.5) | 63 (13.4) | <0.001 |
| Bleeding | 644 (12.5) | 70 (14.9) | 0.13 |
| New pacemaker | 192 (3.7) | 14 (3.0) | 0.4 |
| Indexed EOA, cm^2^/m^2^ | 1.14 (0.96-1.35) | 1.13 (0.95-1.34) | 0.26 |
| THV mean pressure gradient, mmHg | 10.5 (8.0-14.0) | 9.1 (7.0--12.1) | <0.001 |
| PVL ≥moderate | 92 (1.8) | 14 (3.0) | 0.07 |
| 2-year outcomes | | | |
| All-cause mortality within 2 years | 730 (14.2) | 107 (22.7) | <0.001 |
| Cardiovascular mortality within 2 years | 218 (4.2) | 31(6.6) | 0.017 |
| Non-cardiovascular mortality within 2 years | 512 (9.9) | 76 (16.1) | <0.001 |

**Supplemental Table 7. Univariable and multivariable Cox regression analysis of 2-year heart failure death.**

|  | Univariable analysis | | Multivariable analysis | |
| --- | --- | --- | --- | --- |
|  | HR (95% CI) | P value | HR (95% CI) | P value |
| Pre-existing LBBB (vs no LBBB) | 2.70 (1.68-4.35) | <0.001 | 2.22 (1.34-3.68) | 0.0018 |
| New onset LBBB (vs no LBBB) | 0.86 (0.60-1.24) | 0.42 | 0.90 (0.61-1.34) | 0.61 |
| Pre-existing LBBB (vs new onset LBBB) | 3.14 (1.83-5.37) | <0.001 | 2.46 (1.37-4.43) | 0.0025 |
| Age (per 1 yr increase) | 1.04 (1.01-1.08) | 0.0028 | 1.04 (1.01-1.07) | 0.012 |
| Male | 1.64 (1.21-2.22) | 0.0012 | 1.54 (1.10-2.14) | 0.01 |
| Body surface index (per 1m^2^ increase) | 0.36 (0.14-0.89) | 0.026 |  |  |
| NYHA 3 or 4 | 2.50 (1.83-3.39) | <0.001 | 1.38 (0.98-1.94) | 0.063 |
| Dyslipidemia | 0.77 (0.57-1.03) | 0.086 |  |  |
| Diabetes mellitus | 1.64 (1.20-2.23) | 0.0015 | 1.38 (0.99-1.94) | 0.056 |
| Chronic kidney disease | 2.29 (1.53-3.41) | <0.001 | 1.33 (0.87-2.04) | 0.18 |
| Atrial fibrillation | 2.43 (1.78-3.29) | <0.001 | 1.46 (1.04-2.05) | 0.026 |
| COPD | 1.50 (0.97-2.31) | 0.062 |  |  |
| Peripheral artery disease | 2.43 (1.70-3.49) | <0.001 | 1.54 (1.04-2.28) | 0.029 |
| Previous CABG | 3.39 (2.18-5.26) | <0.001 | 2.81 (1.75-4.48) | <0.001 |
| Previous pacemaker | 3.39 (2.03-5.68) | <0.001 | 2.07 (1.19-3.61) | 0.0096 |
| Previous stroke | 1.81 (1.23-2.67) | 0.0026 |  |  |
| Clinical frail score (per 1 group increase) | 1.36 (1.22-1.52) | <0.001 |  |  |
| Non-transfemoral approach | 2.40 (1.65-3.49) | <0.001 |  |  |
| Local anesthesia | 0.48 (0.33-0.69) | <0.001 |  |  |
| Hemoglobin (per 1g/dl increase) | 0.80 (0.73-0.88) | <0.001 |  |  |
| Albumin <3.5g/dl | 2.62 (1.93-3.55) | <0.001 |  |  |
| BNP ≧400 pg/ml or NT-Pro BNP ≧1600 pg/ml | 3.13 (2.28-4.31) | <0.001 | 1.79 (1.25-2.56) | 0.0013 |
| LVEF ≦40% | 3.02 (2.10-4.35) | <0.001 | 1.32 (0.87-2.01) | 0.18 |
| MR ≧moderate | 1.66 (1.11-2.47) | 0.012 |  |  |
| AR ≧moderate | 0.83 (0.49-1.41) | 0.50 |  |  |
| TR ≧moderate | 2.98 (2.05-4.31) | <0.001 | 2.47 (1.65-3.71) | <0.001 |
| Stroke | 0.83 (0.26-2.61) | 0.75 |  |  |
| Myocardial infarction | 6.02 (2.47-14.6) | <0.001 | 4.14 (1.65-10.3) | 0.0023 |
| Vascular complications | 1.53 (0.94-2.49) | 0.086 |  |  |
| AKI | 4.66 (3.30-6.58) | <0.001 | 2.48 (1.67-3.68) | <0.001 |
| Bleeding | 2.89 (2.08-4.11) | <0.001 | 2.00 (1.41-2.85) | 0.001 |
| New pacemaker implantation | 1.40 (0.71-2.74) | 0.32 |  |  |
| PVL ≧moderate | 1.95 (0.86-4.42) | 0.11 | 1.23 (0.49-3.08) | 0.64 |

AKI indicates acute kidney injury; AR, aortic regurgitation; BNP, brain natriuretic peptide; CABG, coronary artery bypass grafting; COPD, chronic obstructive pulmonary disease; LVEF, left ventricular ejection fraction; MR, mitral regurgitation; NYHA, New York Heart Association; PVL, paravalvular leak; and TR, tricuspid regurgitation.

**Supplemental Table 8. Univariable and multivariable Cox regression analysis of sudden cardiac death.**

|  | Univariable analysis | | Multivariable analysis | |
| --- | --- | --- | --- | --- |
|  | HR (95% CI) | P value | HR (95% CI) | P value |
| Pre-existing LBBB (vs no LBBB) | 1.59 (0.48-5.25) | 0.44 | 1.47 (0.44-4.87) | 0.52 |
| New onset LBBB (vs no LBBB) | 1.04 (0.53-2.05) | 0.90 | 1.09 (0.55-2.15) | 0.79 |
| Pre-existing LBBB (vs new onset LBBB) | 1.53 (0.43-5.42) | 0.51 | 1.34 (0.37-4.80) | 0.64 |
| Age (per 1 yr increase) | 1.01 (0.95-1.08) | 0.56 |  |  |
| Male | 0.80 (0.40-1.59) | 0.53 |  |  |
| Body surface index (per 1m^2^ increase) | 0.22 (0.03-1.35) | 0.1 |  |  |
| NYHA 3 or 4 | 1.93 (1.05-3.52) | 0.031 | 1.59 (0.85-2.96) | 0.14 |
| Dyslipidemia | 0.80 (0.44-1.46) | 0.47 |  |  |
| Diabetes mellitus | 1.61 (0.86-2.99) | 0.13 |  |  |
| Chronic kidney disease | 2.01 (0.93-4.33) | 0.074 |  |  |
| Atrial fibrillation | 1.72 (0.90-3.31) | 0.099 |  |  |
| COPD | 1.83 (0.81-4.13) | 0.14 |  |  |
| Peripheral artery disease | 1.39 (0.58-3.30) | 0.45 |  |  |
| Previous CABG | 1.65 (0.51-5.35) | 0.40 |  |  |
| Previous pacemaker | 3.41 (1.22-9.55) | 0.019 |  |  |
| Previous stroke | 0.40 (0.09-1.66) | 0.21 |  |  |
| Clinical frail score (per 1 group increase) | 1.25 (0.99-1.57) | 0.053 |  |  |
| Non-transfemoral approach | 1.61 (0.68-3.81) | 0.27 |  |  |
| Local anesthesia | 1.01 (0.54-1.88) | 0.96 |  |  |
| Hemoglobin (per 1g/dl increase) | 0.90 (0.75-1.08) | 0.29 |  |  |
| Albumin <3.5g/dl | 2.30 (1.24-4.24) | 0.007 | 1.99 (1.06-3.73) | 0.031 |
| BNP ≧400 pg/ml or NT-Pro BNP ≧1600 pg/ml | 1.34 (0.72-2.49) | 0.34 |  |  |
| LVEF ≦40% | 1.78 (0.75-4.22) | 0.18 |  |  |
| MR ≧moderate | 0.61 (0.19-1.99) | 0.42 |  |  |
| AR ≧moderate | 0.89 (0.31-2.50) | 0.83 |  |  |
| TR ≧moderate | 2.26 (1.00-5.09) | 0.047 |  |  |
| Stroke | 1.09 (0.15-7.97) | 0.92 |  |  |
| Myocardial infarction | 4.76 (0.65-34.6) | 0.12 |  |  |
| Vascular complications | 1.34 (0.48-3.76) | 0.57 |  |  |
| AKI | 2.21 (0.93-5.24) | 0.071 |  |  |
| Bleeding | 2.36 (1.19-4.68) | 0.013 | 2.15 (1.08-4.29) | 0.029 |
| New pacemaker implantation | 1.23 (0.29-5.11) | 0.76 |  |  |
| PVL ≧moderate | 2.59 (0.62-10.7) | 0.18 |  |  |

AKI indicates acute kidney injury; AR, aortic regurgitation; BNP, brain natriuretic peptide; CABG, coronary artery bypass grafting; COPD, chronic obstructive pulmonary disease; LVEF, left ventricular ejection fraction; MR, mitral regurgitation; NYHA, New York Heart Association; PVL, paravalvular leak; and TR, tricuspid regurgitation.

**Supplemental Table 9. Comparison of all-cause mortality in each of the two groups**

|  | | Pre-existing vs no LBBB | | New onset vs no LBBB | | Pre-existing vs new onset LBBB | |
| --- | --- | --- | --- | --- | --- | --- | --- |
|  |  | HR (95% CI) | P value | HR (95% CI) | P value | HR (95% CI) | P value |
| All-cause mortality | Unadjusted | 1.44 (1.12-1.87) | 0.0049 | 0.89 (0.77-1.03) | 0.13 | 1.61 (1.22-2.12) | <0.001 |
|  | Multivariable Cox regression analysis | 1.39 (1.06-1.82) | 0.015 | 0.97 (0.83-1.13) | 0.75 | 1.43 (1.07-1.91) | 0.016 |
|  | IPTW analysis | 1.37 (1.02-1.83) | 0.034 | 0.91 (0.78-1.07) | 0.29 | 1.43 (1.04-1.97) | 0.025 |

CI indicates confidence interval; HR, hazard ratio; IPTW, inverse probability treatment weighting; and LBBB, left bundle branch block.

**Supplemental Figure 1. Proportional hazard assumptions of each outcome**

**
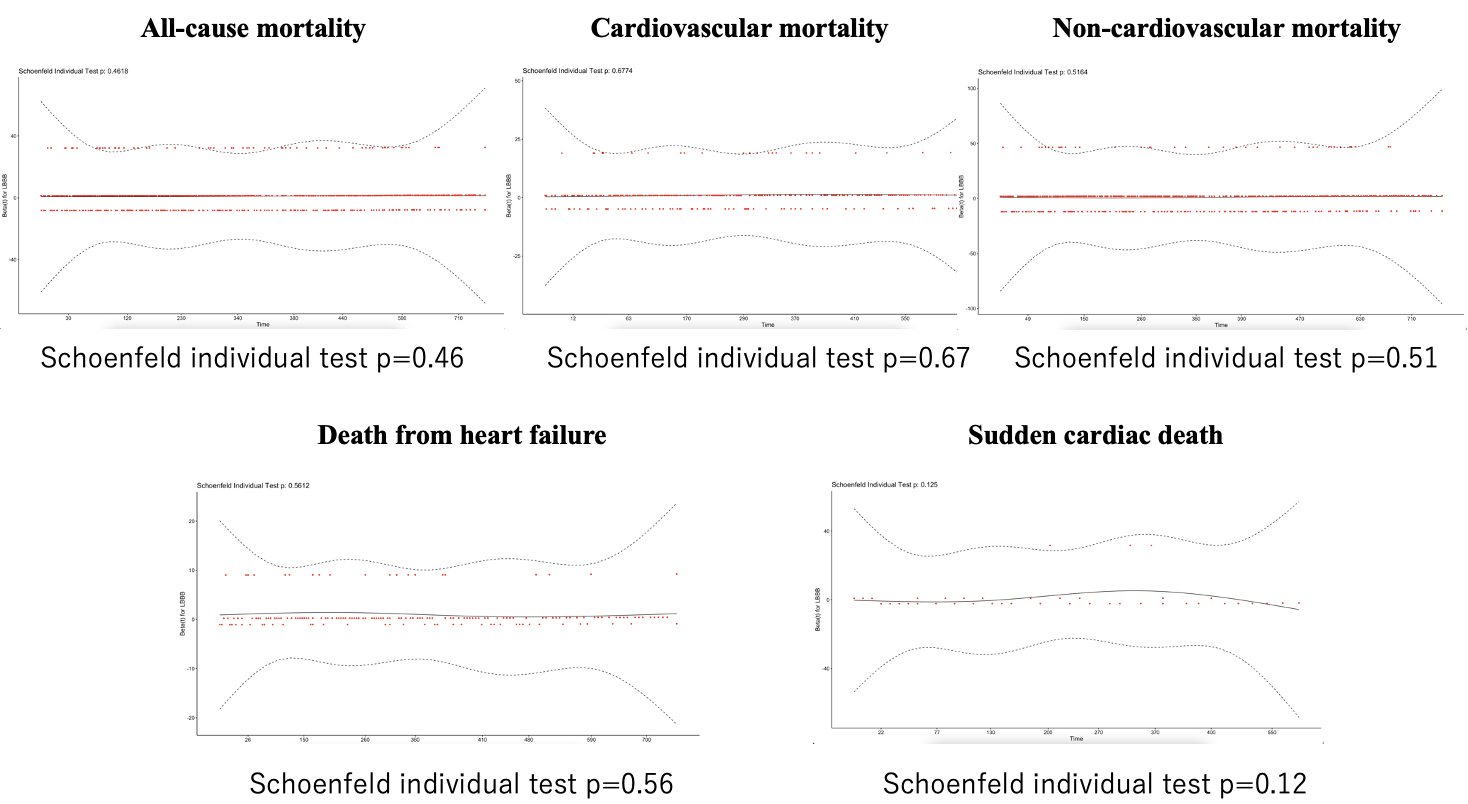
**

Plots of Schoenfeld residuals and Schoenfeld individual tests.

**Supplemental Figure 2. Pattern of missing values**

**
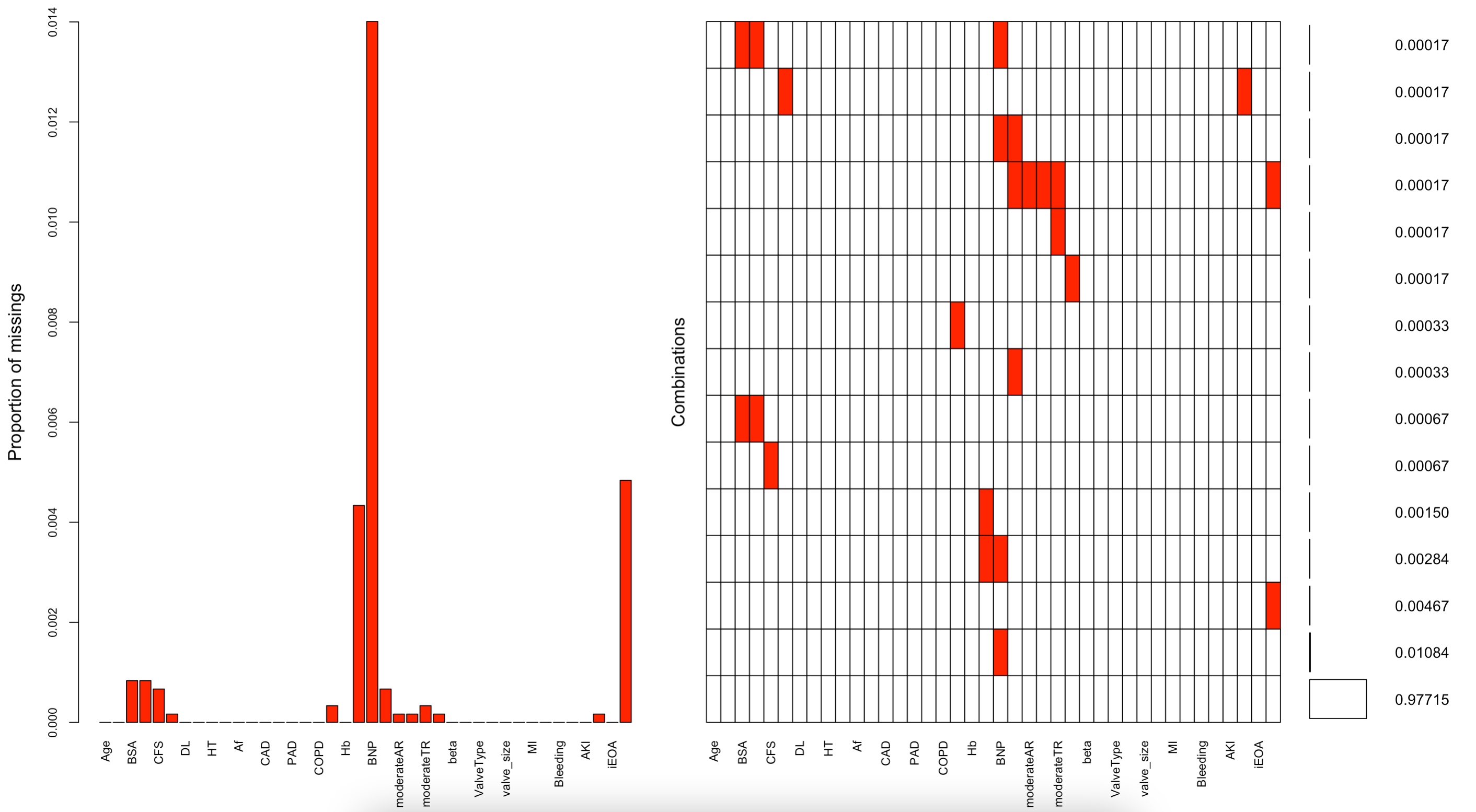
**

Percentage and pattern analysis of missing values.

**Supplemental Figure 3. Subgroup analyses for all-cause mortality**

**
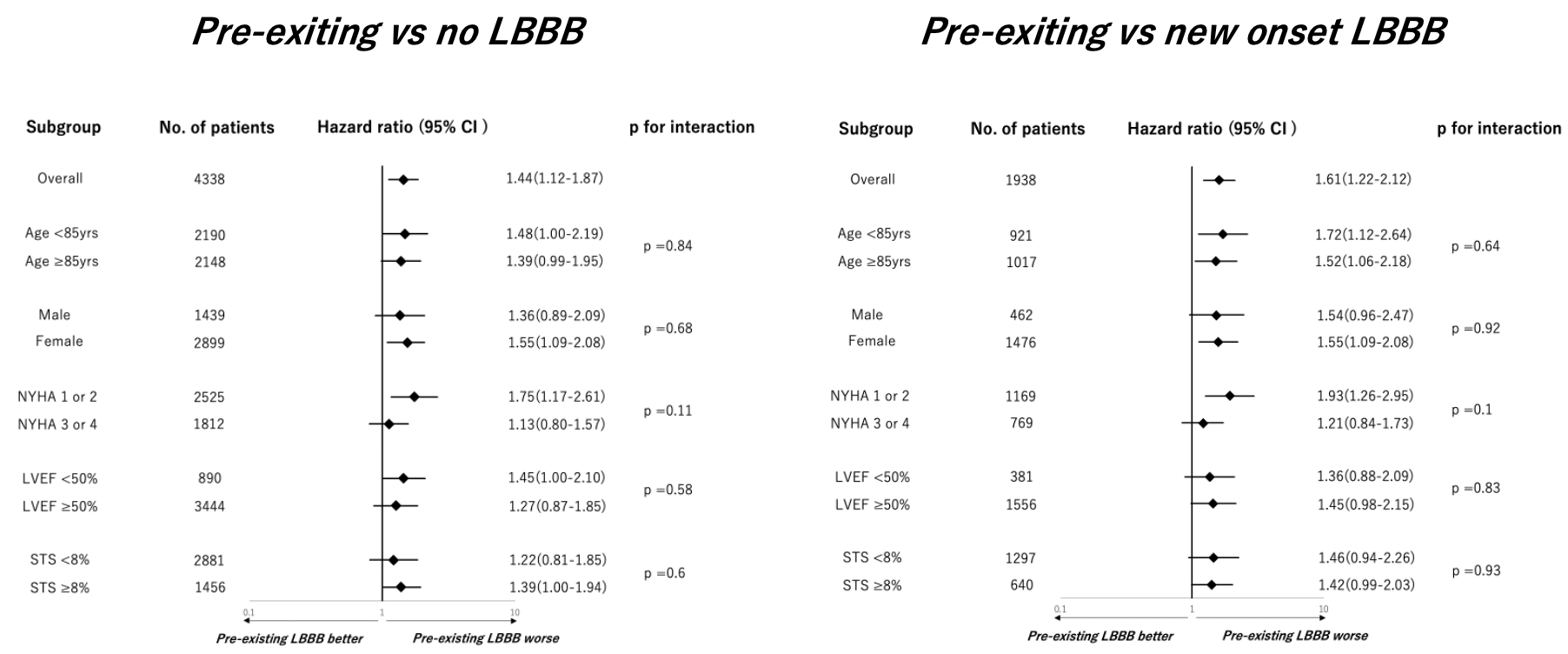
**

Forrest plot representing the hazard ratios of 2-year all-cause mortality in patients with pre-existing LBBB compared to patients without, stratified by pre-procedural characteristics.

HR indicates hazard ratio; LVEF, left ventricular ejection fraction; NYHA, New York Heart Association; STS, Society of Thoracic Surgeons; and LBBB, left bundle branch block.

**Supplemental Figure 4. Sensitivity analyses**

**
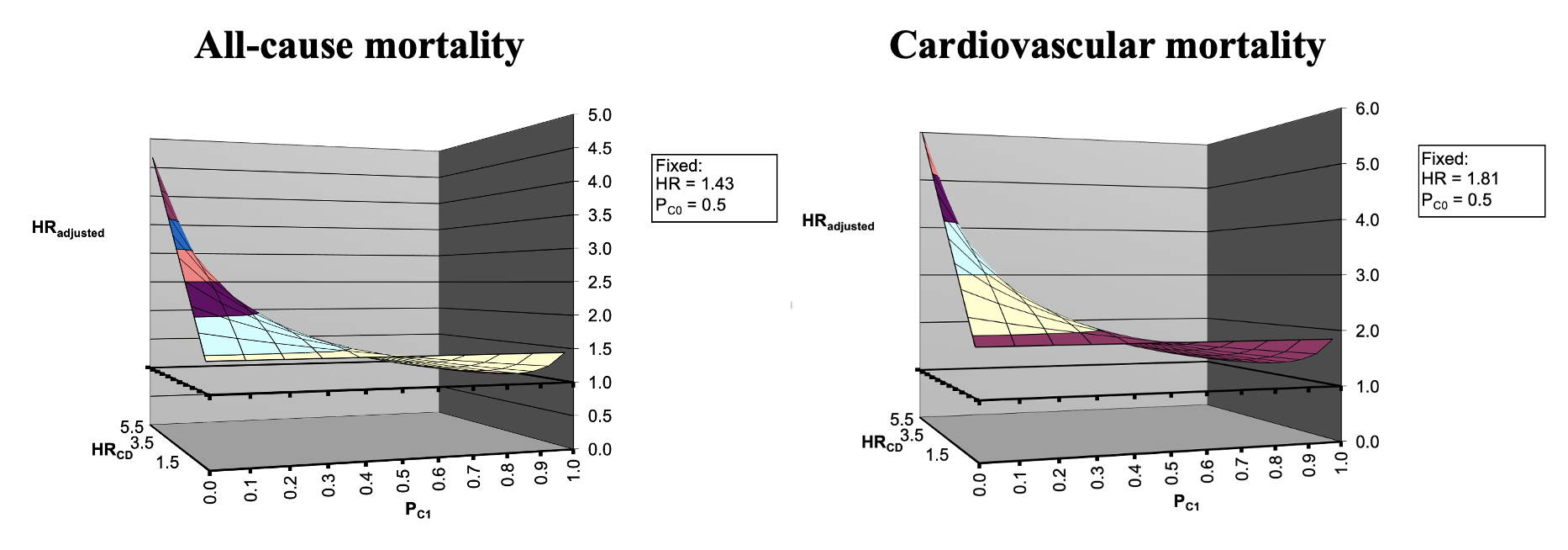
**

The observed hazard ratios for pre-existing LBBB using new onset LBBB as reference were 1.43 for all-cause mortality and 1.81 for cardiovascular mortality. Prevalence of unmeasured confounders in the new onset LBBB group was set at 0.50 (P_C0_). Two factors were varied: the strength of the unmeasured confounder-outcome association (HR_CD_, 1.0–5.5) and the prevalence of the unmeasured confounder in the pre-existing LBBB group (P_C1_, 0.0–1.0).

HR indicates hazard ratio; and LBBB, left bundle branch block.
